# Supplementary material for: Comparing mutational pathways to lopinavir resistance in HIV-1 subtypes B versus C
Source: PLoS Comput Biol. 2021 Sep 7;17(9):e1008363. doi: 10.1371/journal.pcbi.1008363 (PMC8448360; doi:10.1371/journal.pcbi.1008363)
Supplement: S1 Text — (PDF) [file pcbi.1008363.s001.pdf]

S1 Text. Additional notes on the model and parameter estimation

The probability density of the waiting time T_j conditioned on the times to mutation of its parents $\mathrm{pa}(j)$ is defined as

$$f_T\left(t_j \mid (t_u)_{u \in \mathrm{pa}(j)}; \lambda_j\right) = \lambda_j \exp\left[-\lambda_j (t_j - \max_{u \in \mathrm{pa}(j)} t_u)\right] \mathbb{I}(t_j \geq \max_{u \in \mathrm{pa}(j)} t_u), \tag{1}$$

The likelihood function for N independent, observed genotypes $\mathcal{Y} = (Y^{(1)}, \ldots, Y^{(N)})$ is

$$\mathcal{L}_{\mathcal{Y}}(\lambda, \epsilon, P) = \prod_{i=1}^{N} \Pr\left(Y^{(i)}; \lambda, \epsilon, P\right), \tag{2}$$

where $\lambda = (\lambda_1, \ldots, \lambda_p)$ and the probability of each observed genotype $Y^{(i)}$ is

$$\Pr\left(Y^{(i)}; \lambda, \epsilon, P\right) = \sum_{X \in J(P)} \Pr\left(Y^{(i)} \,\middle|\, X\right) \Pr\left(X; \lambda, \epsilon, P\right). \tag{3}$$

The probability of observing genotype Y given the true underlying genotype X is

$$\Pr(Y \mid X) = \epsilon^{d_H(X,Y)} (1-\epsilon)^{p - d_H(X,Y)}, \tag{4}$$

where ϵ is the per-locus error probability and d_H is the Hamming distance.
The probability of the true genotype X is defined in terms of the waiting times,

$$\Pr(X; \lambda, \epsilon, P) = \int_{\mathbb{R}^{p+1}_{\geq 0}} \left(\prod_{j=1}^{p} f_Z(z_j; \lambda_j)\right) f_Z(z_s; \lambda_s)\, \mathbb{I}(z, z_s \vdash X)\, \mathrm{d}z, \tag{5}$$

where the indicator function $\mathbb{I}$ encodes mutation times that can give rise to genotype X, i.e., $z, z_s \vdash X$, $z_j = t_j - \max_{u \in \mathrm{pa}(j)} t_u$ $(j = 1, \ldots, p)$, and $f_Z(z_j; \lambda_j) = f_T\left(t_j \mid (t_u)_{u \in \mathrm{pa}(j)}; \lambda_j\right)$. Henceforth, we also set $z = (z_1, \ldots, z_p, z_s)$.

E-step The expected value of the complete-data log-likelihood $\ell_{\mathcal{X},\mathcal{Z},\mathcal{Y},}(\lambda, \epsilon)$ with respect to the current conditional distribution of the hidden data (i.e., the unobserved true genotypes $\mathcal{X} = (X^{(1)}, \ldots, X^{(N)})$ and mutation times $\mathcal{Z} = (Z^{(1)}, \ldots, Z^{(N)})$), given the observed genotypes $\mathcal{Y}$, as well as the current estimates of the parameters $\lambda^{(k)}$ and $\epsilon^{(k)}$ is given by

$$\mathbb{E}_{\mathcal{X},\mathcal{Z}|(\mathcal{Y},\lambda^{(k)},\epsilon^{(k)})}\left[\ell_{\mathcal{Y},\mathcal{X},\mathcal{Z}}(\lambda,\epsilon)\right] = \sum_{x^{(1)} \in J(P)} \cdots \sum_{x^{(N)} \in J(P)} \int_{\mathbb{R}^{p+1}_{\geq 0}} \cdots \int_{\mathbb{R}^{p+1}_{\geq 0}} \prod_{i=1}^{N} f_{X,Z}\left(x^{(i)}, z^{(i)} \,\middle|\, Y = y^{(i)}; \lambda^{(k)}, \epsilon^{(k)}\right) \ell_{\mathcal{Y},\mathcal{X},\mathcal{Z}}(\lambda,\epsilon)\, \mathrm{d}z^{(1)} \cdots \mathrm{d}z^{(N)}, \tag{6}$$

where k denotes the current MCEM iteration. Assuming independent observations the complete-data log-likelihood is

$$\ell_{\mathcal{X},\mathcal{Z},\mathcal{Y},}(\lambda, \epsilon) = \sum_{i=1}^{N} \left[\log \Pr\left(Y = y^{(i)} \mid X = x^{(i)}\right) + \log f_Z\left(z^{(i)}\right)\right], \tag{7}$$

where the probability of observing genotype Y given the true underlying genotype X is

$$\Pr\left(Y \mid X\right) = \epsilon^{d_H(X,Y)} \left(1-\epsilon\right)^{p-d_H(X,Y)}, \tag{8}$$

where ϵ is the per-locus error probability and d_H is the Hamming distance.
According to Bayes' theorem,

$$f_{X,Z}\left(x, z \,\middle|\, Y = y; \lambda^{(k)}, \epsilon^{(k)}\right) = \frac{\Pr\left(Y = y \mid X = x;\, \epsilon^{(k)}\right) f_Z(z;\, \lambda^{(k)}) \mathbb{I}\left(z \vdash X\right)}{\Pr\left(Y = y; \lambda^{(k)}, \epsilon^{(k)}\right)}. \tag{9}$$

We denote the numerator of Eq. (9) by $A^{(k)}\left(x, y, z\right)$ to obtain the expected complete-data log-likelihood

$$\begin{aligned}
&\mathbb{E}_{\mathcal{X},\mathcal{Z}|(\mathcal{Y},\lambda^{(k)},\epsilon^{(k)})}\left[\ell_{\mathcal{Y},\mathcal{X},\mathcal{Z}}\left(\lambda, \epsilon\right)\right] = \\
&\quad \sum_{i=1}^{N} \sum_{x^{(i)} \in J(P)} \int_{\mathbb{R}_{\geq 0}^{p+1}} \frac{A^{(k)}\left(x^{(i)}, y^{(i)}, z^{(i)}\right)}{\Pr\left(Y = y^{(i)}; \lambda^{(k)}, \epsilon^{(k)}\right)} \left[\log \Pr\left(Y = y^{(i)} \mid X = x^{(i)}\right) + \right. \\
&\qquad \left. \log f_Z\left(z^{(i)}\right)\right] \mathrm{d}z^{(i)}.
\end{aligned} \tag{10}$$

M-step Here, we maximize Eq. (10) with respect to the parameters ϵ and λ_j, $j = 1, \ldots, p$, which yields

$$\hat{\epsilon}^{(k)} = \frac{1}{N} \sum_{i=1}^{N} \frac{\sum_{x^{(i)} \in J(P)} \int_{\mathbb{R}_{\geq 0}^{p+1}} A^{(k)}\left(x^{(i)}, y^{(i)}, z^{(i)}\right) \frac{1}{p} d_H(x^{(i)}, y^{(i)}) \mathrm{d}z^{(i)}}{\sum_{x^{(i)} \in J(P)} \int_{\mathbb{R}_{\geq 0}^{p+1}} A^{(k)}\left(x^{(i)}, y^{(i)}, z^{(i)}\right) \mathrm{d}z^{(i)}}. \tag{11}$$

$$\hat{\lambda}_j^{(k)} = \left[\frac{1}{N} \sum_{i=1}^{N} \frac{\sum_{x^{(i)} \in J(P)} \int_{\mathbb{R}_{\geq 0}^{p+1}} A^{(k)}\left(x^{(i)}, y^{(i)}, z^{(i)}\right) z_j^{(i)} \mathrm{d}z^{(i)}}{\sum_{x^{(i)} \in J(P)} \int_{\mathbb{R}_{\geq 0}^{p+1}} A^{(k)}\left(x^{(i)}, y^{(i)}, z^{(i)}\right) \mathrm{d}z^{(i)}}\right]^{-1}. \tag{12}$$
