# Supplementary material for: Comparing mutational pathways to lopinavir resistance in HIV-1 subtypes B versus C
Source: PLoS Comput Biol. 2021 Sep 7;17(9):e1008363. doi: 10.1371/journal.pcbi.1008363 (PMC8448360; doi:10.1371/journal.pcbi.1008363)
Supplement: S2 Text — (PDF) [file pcbi.1008363.s002.pdf]

S2 Text. Additional details on the assessment of importance sampling schemes on simulated data

When estimating the error rate ϵ, we observe that the forward sampling schemes tend to overestimate small error rates, while the backward sampling schemes tend to underestimate high error rates. In all cases, the variance of the estimated error rate decreases as the number of mutations increases and, in most cases, $\hat{\epsilon}$ converges to the true value. Recalling that the error rate is defined per locus, with an increase in the number of mutations and in the number of genotypes, we have more power to estimate ϵ. As an exception, the estimates obtained by the Bernoulli sampling deteriorate as the number of mutations increases, because most sampled candidate genotypes have weight zero. In fact, the Bernoulli sampling scheme failed to provide any samples for the data sets with 5% error rate and 256 mutations, as well as for the data sets with 10% error rate and 128 and 256 mutations.

By contrast, the relative error in estimating the rate parameters λ increases with the number of mutations and sampling schemes tend to err towards underestimating the rate parameters. This is likely due to the bounded number of genotypes to 1000 for data sets with more than 32 mutations, combined with the density of the simulated networks (Fig S8). For this particular constellation of sample size N and number L of samples drawn from the proposal distribution, for posets with more than 32 mutations the sampling schemes fall short of accurately estimating the rate parameters.

We also evaluate the Hamming k-neighborhood sampling for different k (Fig S6E). We observe that as the number of mutations increases, we need to expand the neighborhood based on the Hamming distance for accurate estimation of the model parameters. However, the run time increases substantially and becomes a limiting factor for larger posets (Fig S9).

Once again, the performance of the Bernoulli sampling scheme declines as the number of mutations increases, and for data sets with more than 64 mutations, the relative median error for the rate parameters is outside the range displayed in Fig S7.

From these simulation studies, we conclude that the best performing schemes are the forward and backward-AR sampling schemes.
