# Supplementary figures and images for: Comparing mutational pathways to lopinavir resistance in HIV-1 subtypes B versus C
Source: PLoS Comput Biol. 2021 Sep 7;17(9):e1008363. doi: 10.1371/journal.pcbi.1008363 (PMC8448360; doi:10.1371/journal.pcbi.1008363)

**A**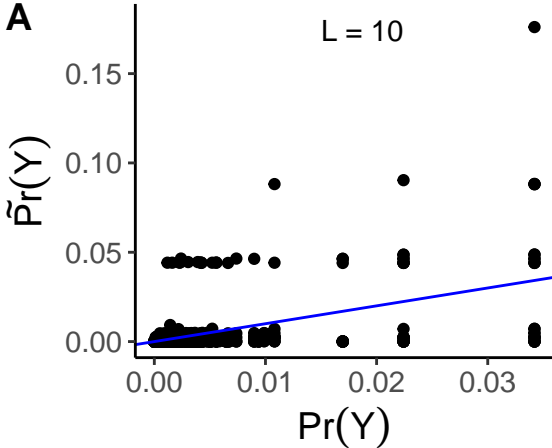**B**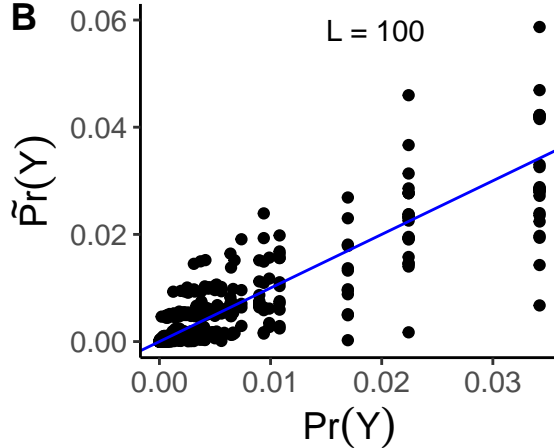**C**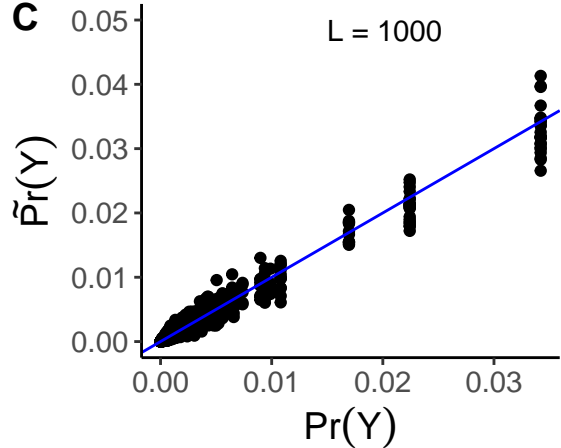

Supplement: S1 Fig — Probability of the observed genotype estimated by using the forward sampling scheme Pr˜(Y=y) (y-axis, Eq 8) vs. the exact solution Pr(Y = y) (x-axis). The data set consists of N = 800 genotypes with p = 16 mutations and an error rate of 5%. Results are obtained by drawing A L = 10, B L = 100, and C L = 1000 samples from the proposal distribution. (PDF) [file pcbi.1008363.s003.pdf]

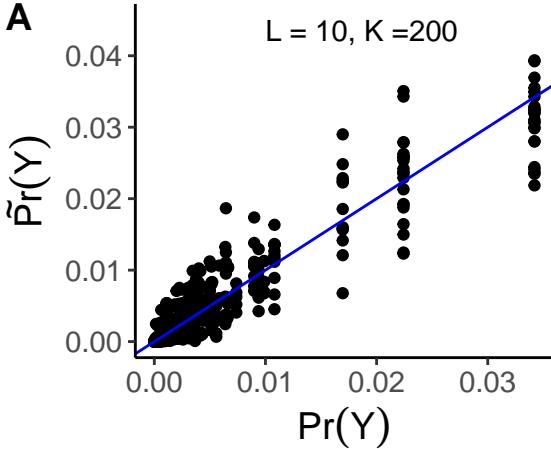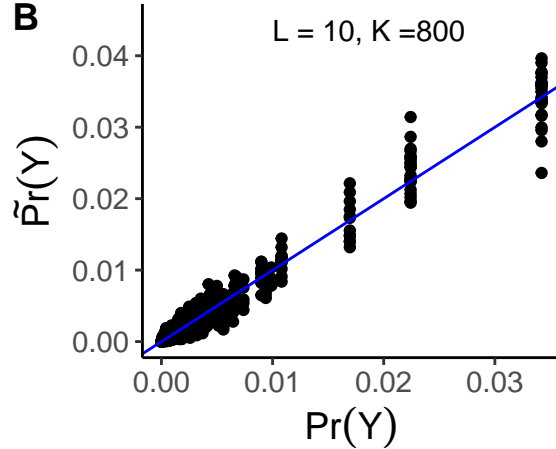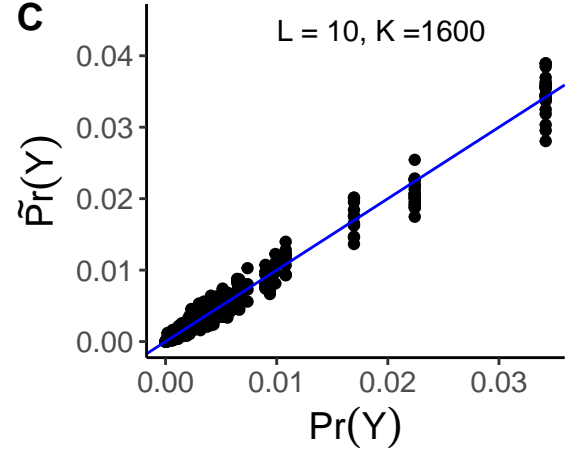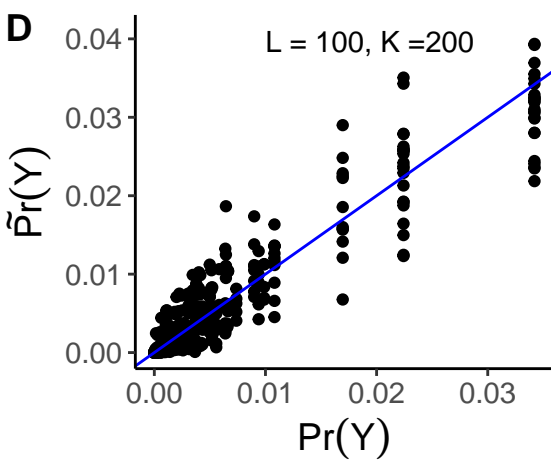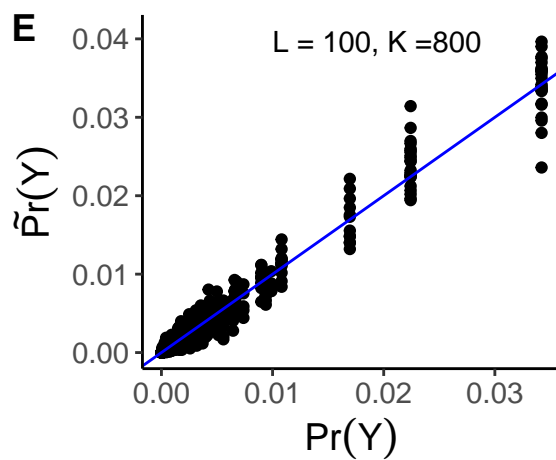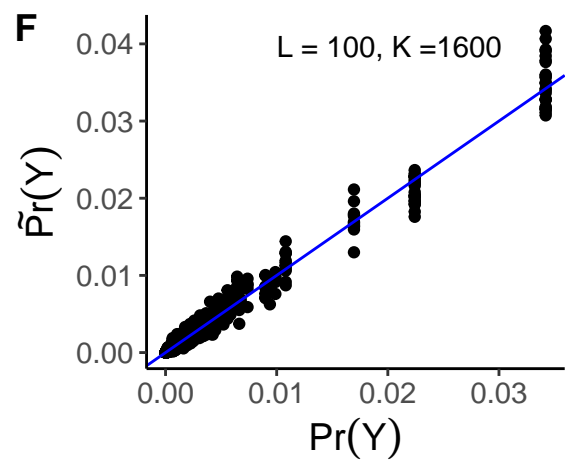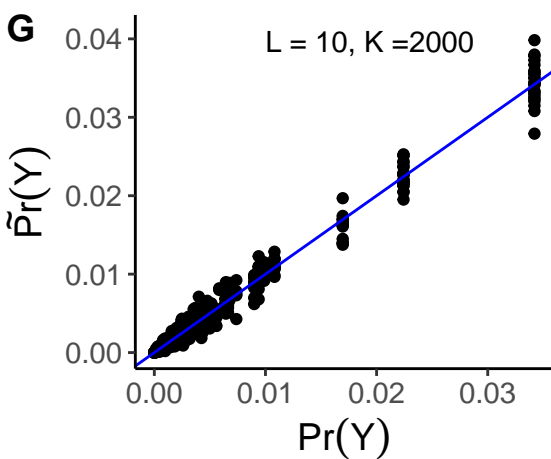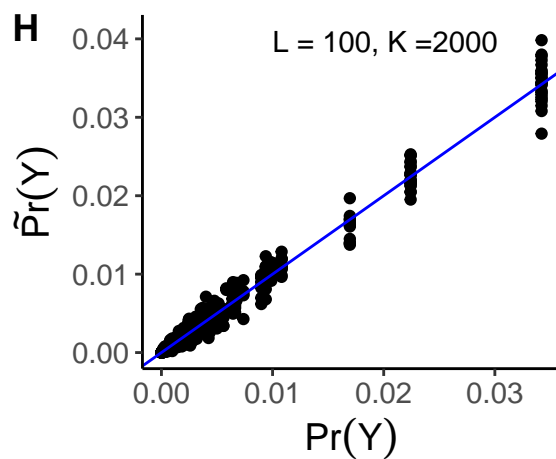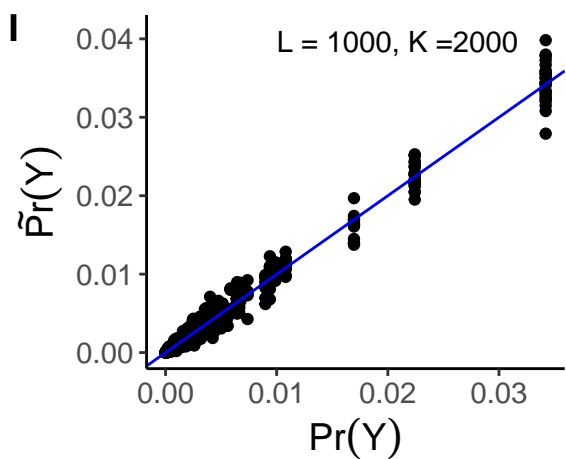

Supplement: S2 Fig — Probability of the observed genotype estimated by using the forward-pool sampling scheme Pr˜(Y=y) (y-axis, Eq 8) vs. the exact solution Pr(Y = y) (x-axis). The data set consists of N = 800 genotypes with p = 16 mutations and an error rate of 5%. First, we evaluate the impact of the size of the initial pool on the accuracy of the approximations. We show results for pools consisting of A, D K = 200, B, E K = 800, and C, F K = 1600 samples, while the number of samples drawn from the proposal distribution is set to either A-C L = 10 or D-F L = 100. Next, we evaluate the impact of the number of samples drawn from the proposal distribution (G L = 10; H L = 100; I L = 1000), while the size of the initial pool is kept constant at K = 2000 samples. We observe that the accuracy of the computation improves primarily as the size of the initial pool increases. By default, the size of the initial pool of waiting times is set to K = p × L. (PDF) [file pcbi.1008363.s004.pdf]

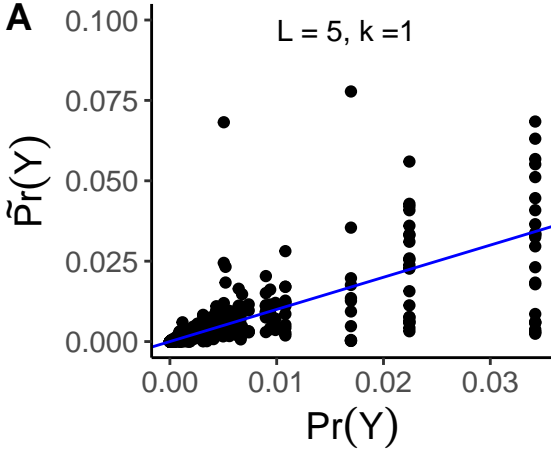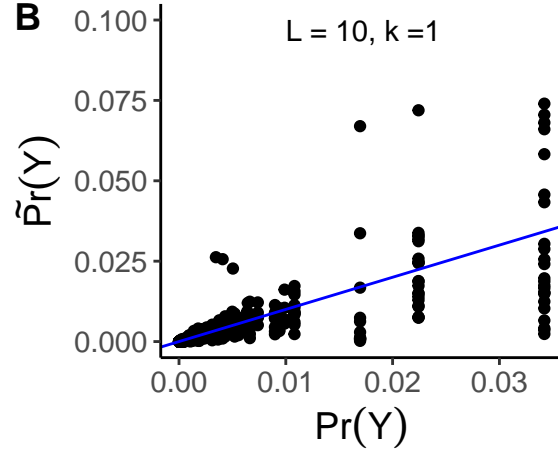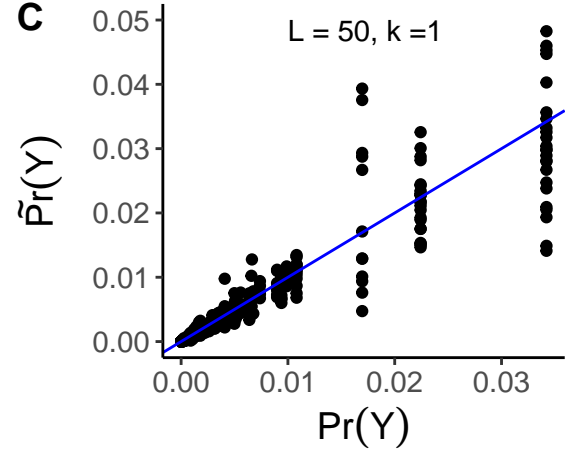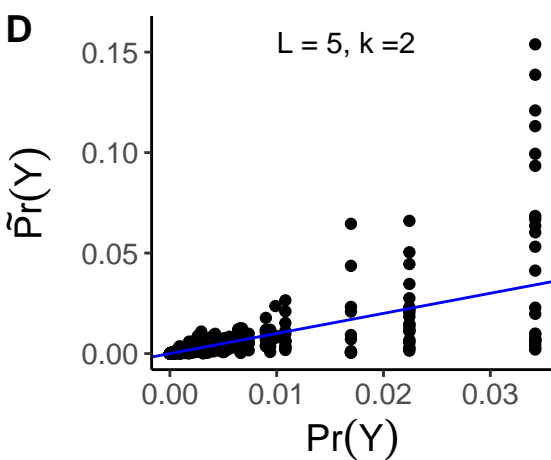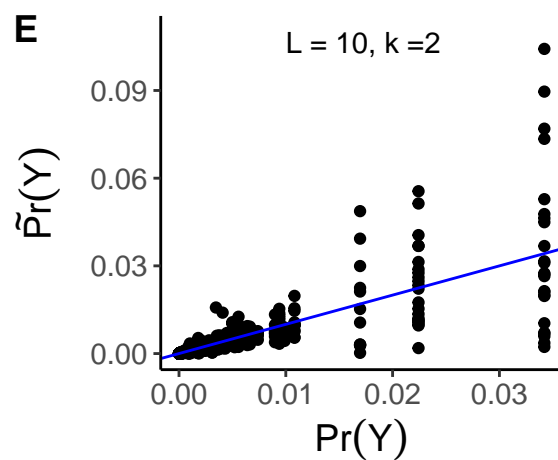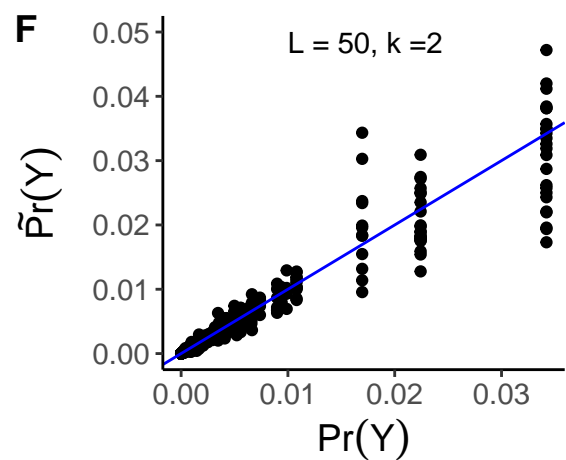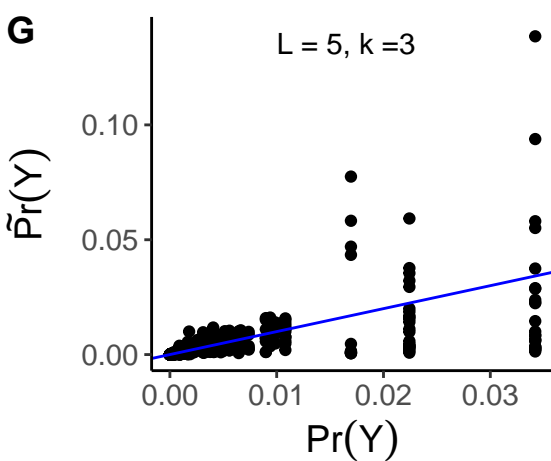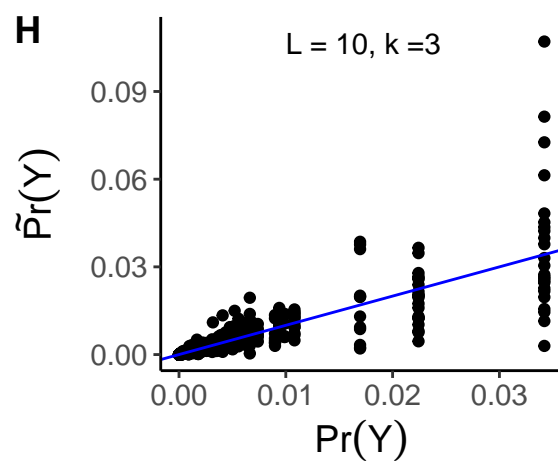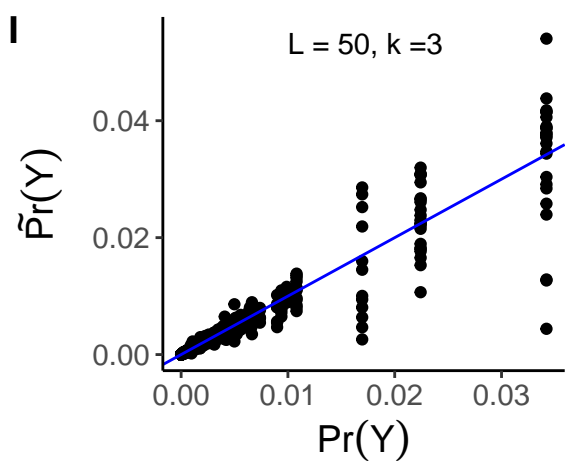

Supplement: S3 Fig — Probability of the observed genotype estimated by using the Hamming k-neighborhood sampling scheme Pr˜(Y=y) (y-axis, Eq 8) vs. the exact solution Pr(Y = y) (x-axis). The data set consists of N = 800 genotypes with p = 16 mutations and an error rate of 5%. Results are shown for A-C a neighborhood including the leading and the first-order terms (k = 1), D-F a neighborhood including the leading, the first-order, and the second-order terms (k = 2), and G-I a neighborhood including the leading, the first-order, the second-order, and the third-order terms (k = 3). In this case, the value of L indicates the number of waiting time vectors sampled per genotype in the neighborhood. (PDF) [file pcbi.1008363.s005.pdf]

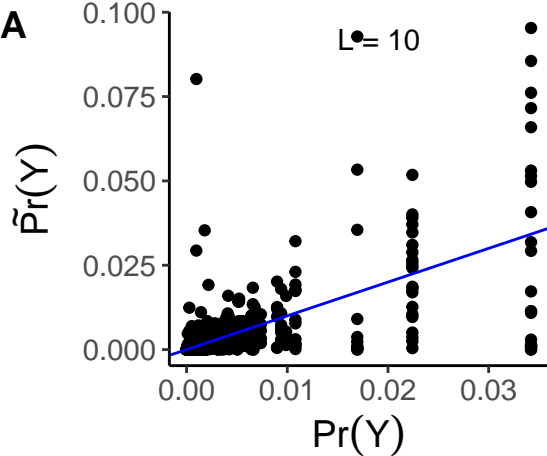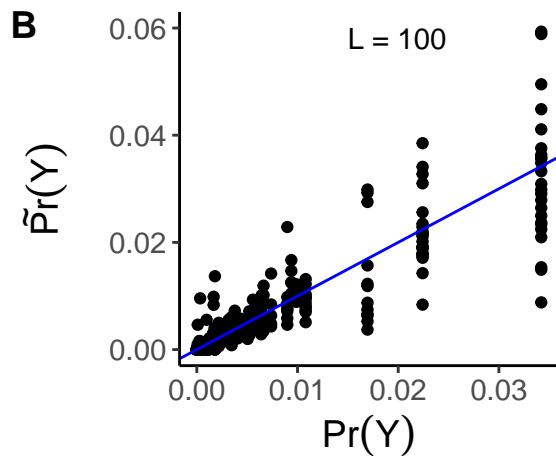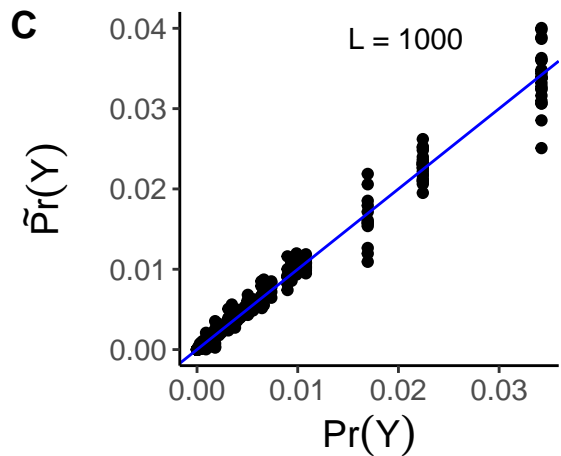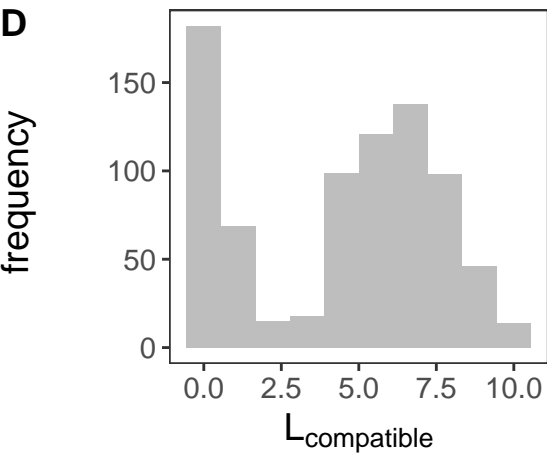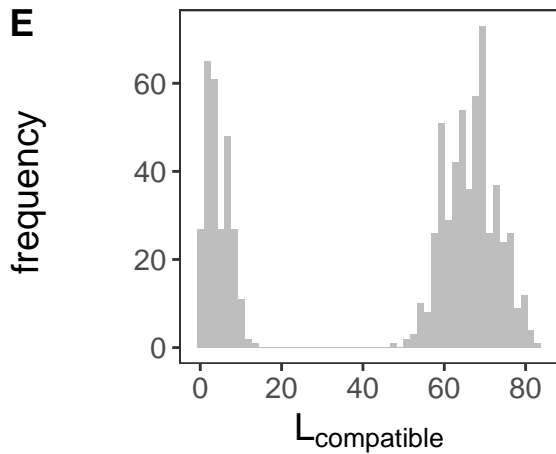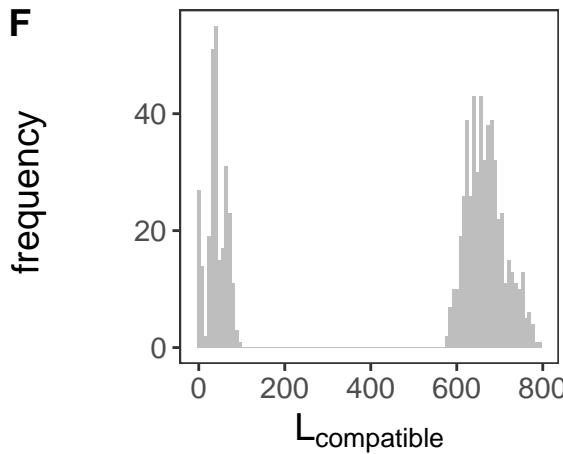

Supplement: S4 Fig — Probability of the observed genotype estimated by using the Bernoulli sampling scheme Pr˜(Y=y) (y-axis, Eq 8) vs. the exact solution Pr(Y = y) (x-axis). The data set consists of N = 800 genotypes with p = 16 mutations and an error rate of 5%. Results are obtained by drawing A L = 10, B L = 100, and C L = 1000 samples from the proposal distribution. In the lower panel, we show the number of samples compatible with the poset Lcompatible per genotype for D L = 10, E L = 100, and F L = 1000 samples drawn from the proposal distribution. (PDF) [file pcbi.1008363.s006.pdf]

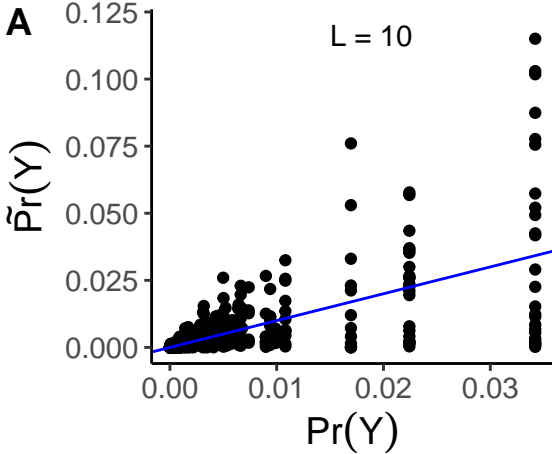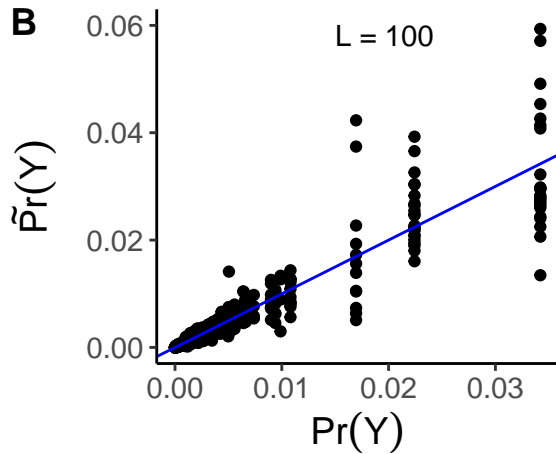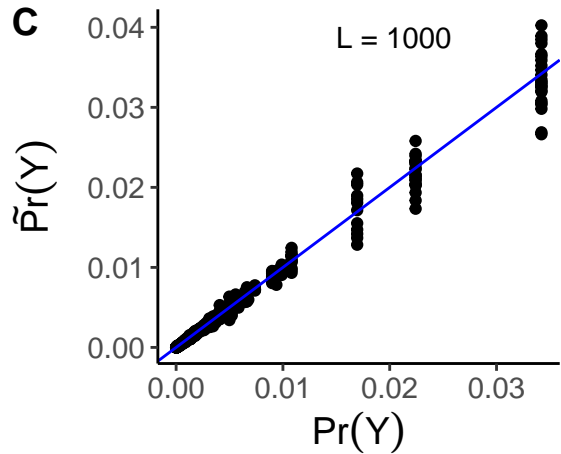

Supplement: S5 Fig — Probability of the observed genotype estimated by using the backward-AR sampling scheme Pr˜(Y=y) (y-axis, Eq 8) vs. the exact solution Pr(Y = y) (x-axis). The data set consists of N = 800 genotypes with p = 16 mutations and an error rate of 5%. Results are obtained by drawing A L = 10, B L = 100, and C L = 1000 samples from the proposal distribution. (PDF) [file pcbi.1008363.s007.pdf]

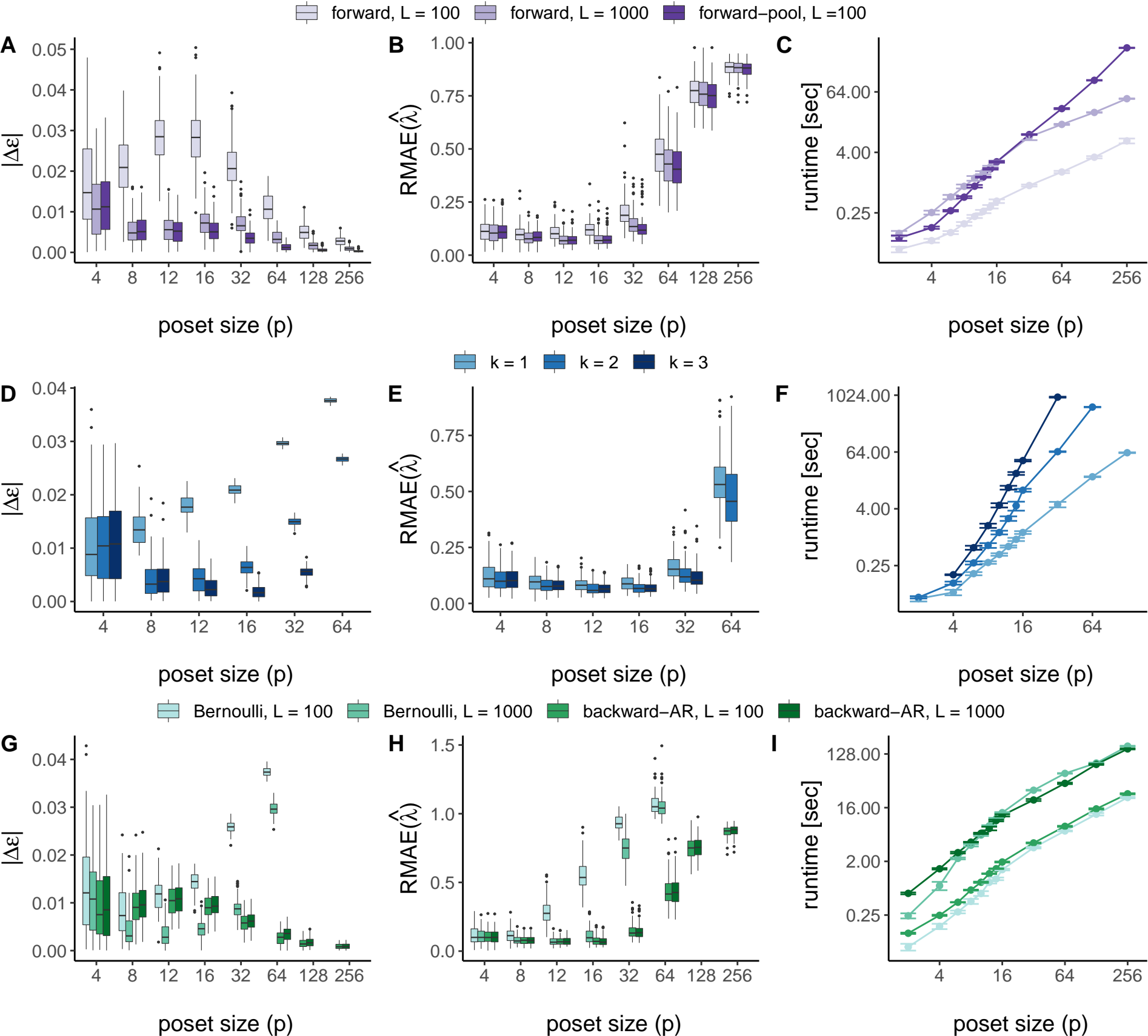

Supplement: S6 Fig — Box plots of the absolute error in estimating the error rate ϵ^ for the true poset P by using A the forward sampling, D the Hamming k-neighborhood sampling, and G the Bernoulli or the backward-AR sampling. Box plots of the relative median absolute error (RMAE) for the estimated rate parameters λ^ by using B the forward sampling, E the Hamming k-neighborhood sampling, and H the Bernoulli or the backward-AR sampling. Average run times over simulated data sets for C the forward sampling, F the Hamming k-neighborhood sampling, and I the Bernoulli or the backward-AR sampling. Results correspond to 100 simulated data sets for each of the number of mutations and 100 iterations of the MCEM algorithm. The number of samples drawn from the proposal distribution is L = 10, 100, 1000, as shown in the corresponding legend. The sample size is N = min(50 p, 1000) and the true error rate is ϵ = 0.05. (PDF) [file pcbi.1008363.s008.pdf]

forward
  Hamming 3-neighborhood, L=10
  backward-AR

forward-pool, L=100
  Bernoulli
  H-CBN

**A**

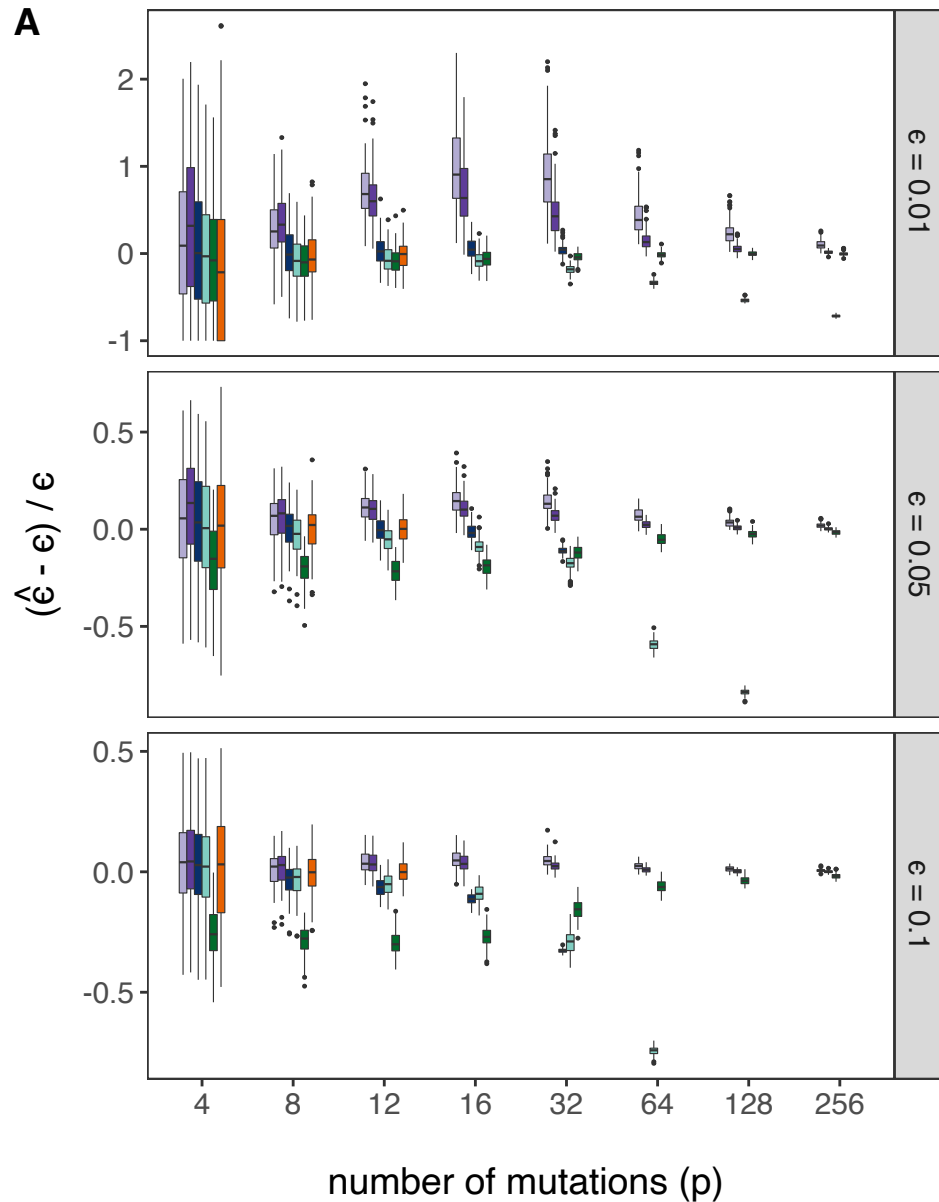

**B**

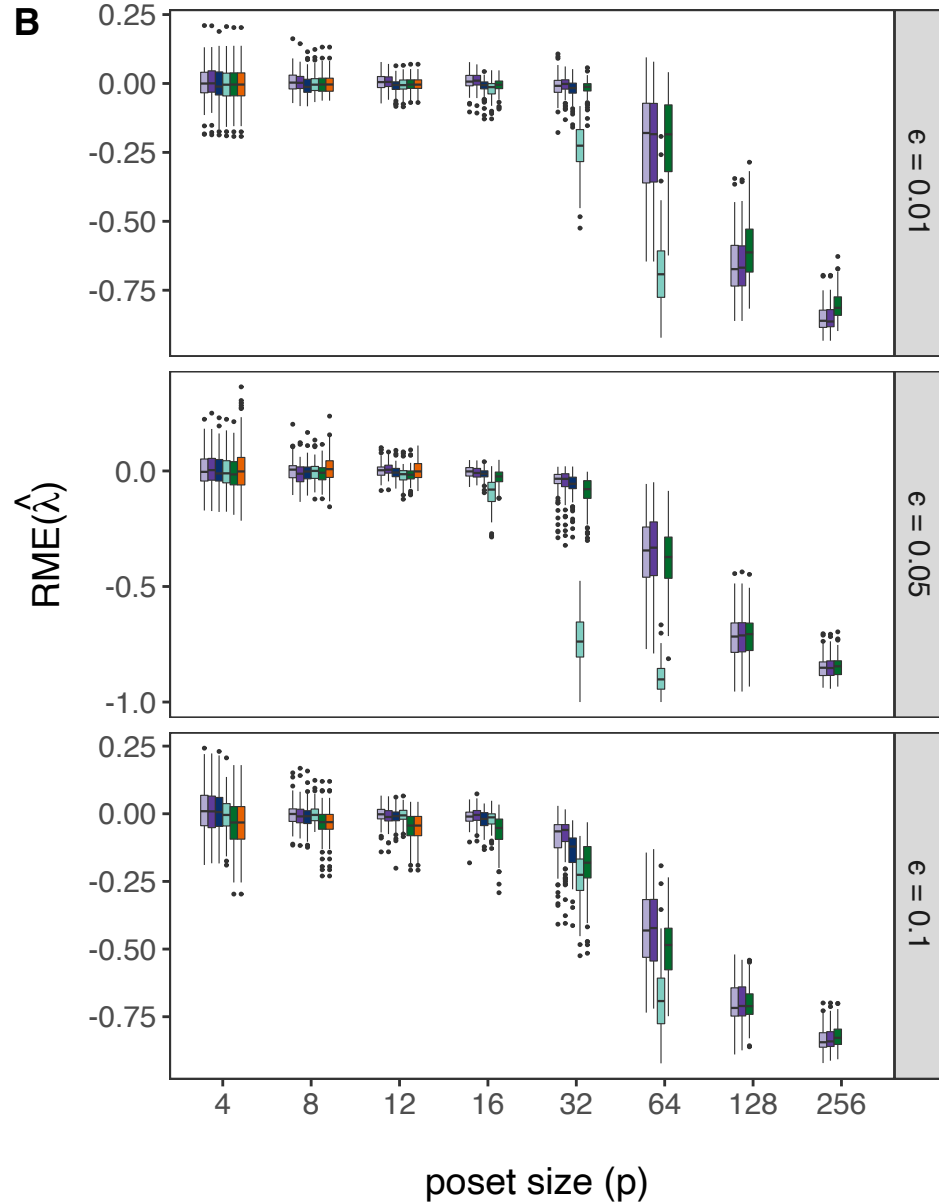

Supplement: S7 Fig — A Box plots of the relative difference between true (ϵ) and estimated (ϵ^) error rate (y-axis) for 100 simulated data sets for each of the evaluated model sizes (x-axis). B Box plots of the relative median error (RME; y-axis) of the estimated rate parameters λ^. The relative (median) error is given by median(λ^-λ)median(λ). Different colors indicate different importance sampling schemes. The sample size is N = min(50 p, 1000) and the number of samples drawn from the proposal distribution is set to L = 1000 unless specified otherwise. We run 100 iterations of the Monte Carlo EM algorithm. (PDF) [file pcbi.1008363.s009.pdf]

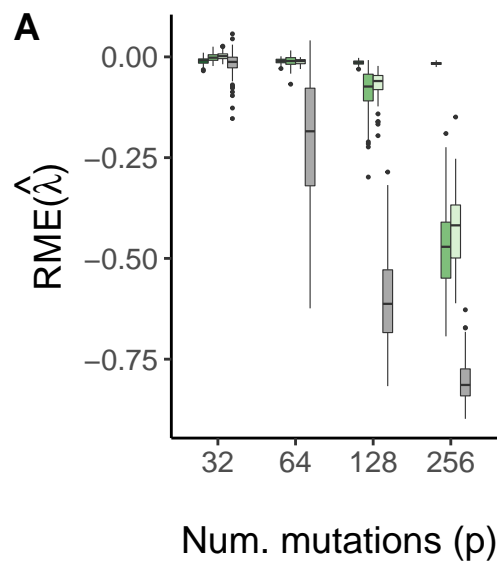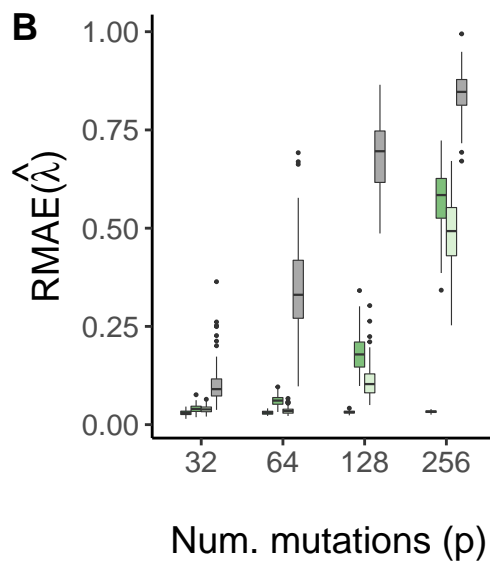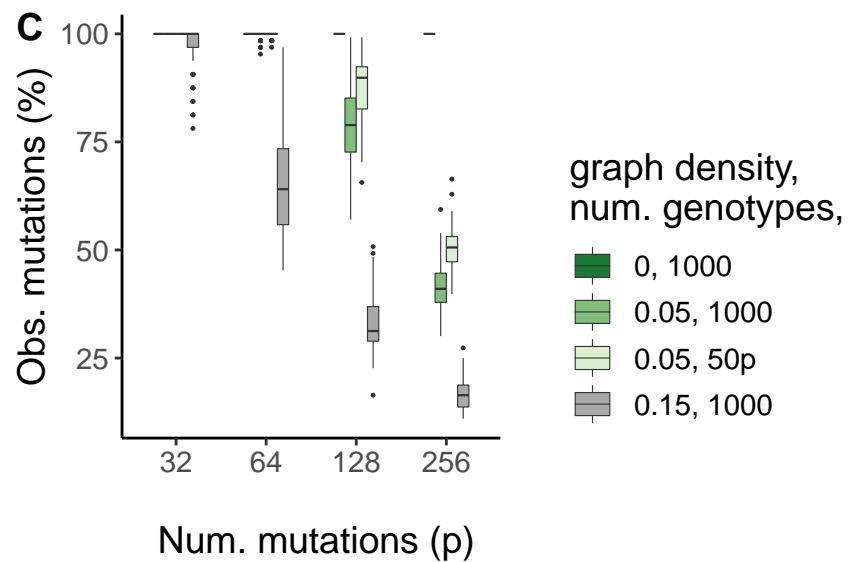

Supplement: S8 Fig — Box plots of A the relative median error (RME) and B the relative median absolute error (RMAE) for the estimated rate parameters λ^, while varying the numbers of order constraints (S1 Table) and mutations, as well as varying the number of simulated genotypes as indicated in this figure legend. C Percentage of mutations marginally observed in the true underlying genotypes. For these mutations, there is evidence in the data to estimate the corresponding rate parameters. Each box plot shows results for 100 different simulated data sets. The number of order constrains, or equivalently, the number of edges in the simulated networks is depicted as graph density, were 0 means independent mutations and 1 corresponds to a linear chain. (PDF) [file pcbi.1008363.s010.pdf]

10 30 50

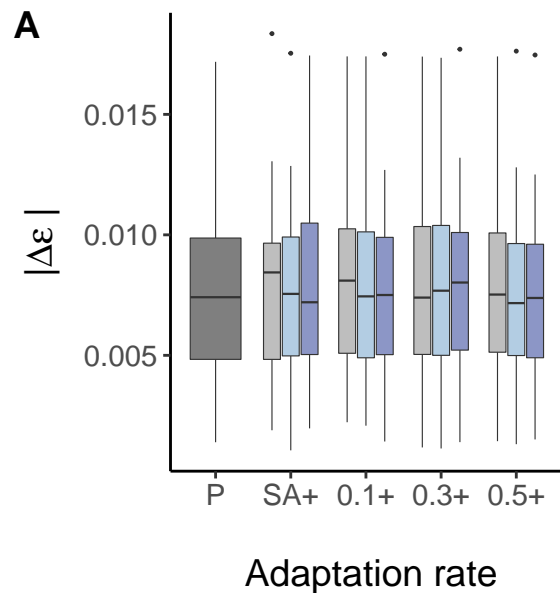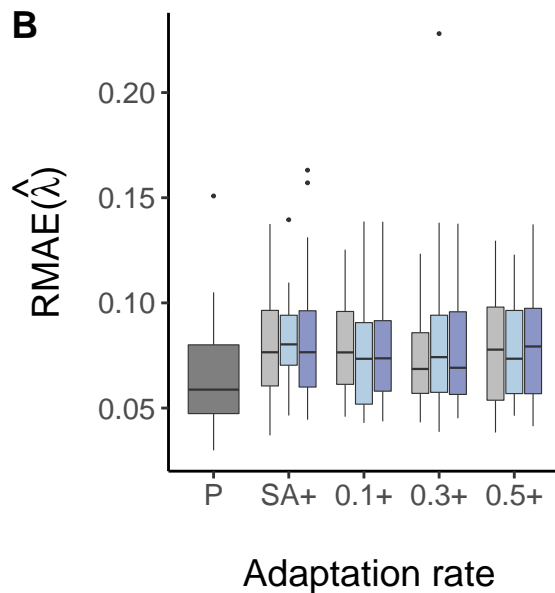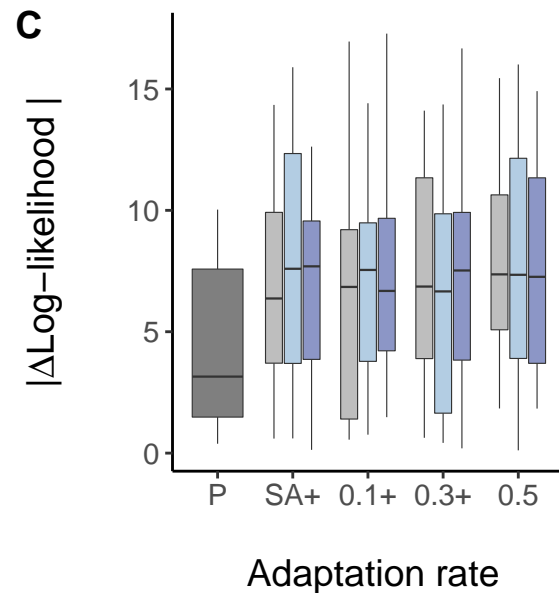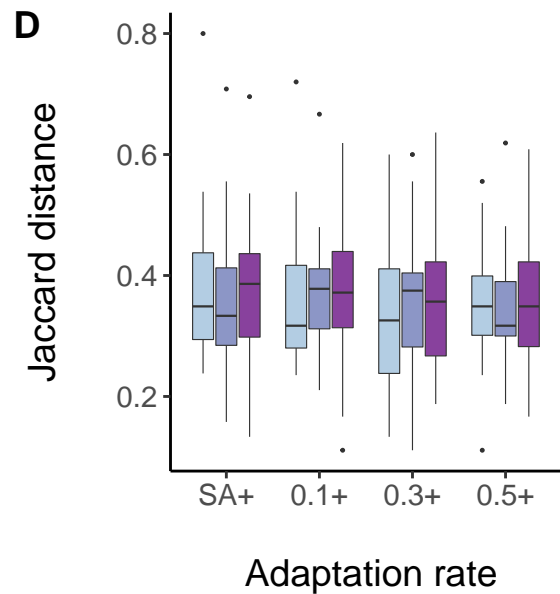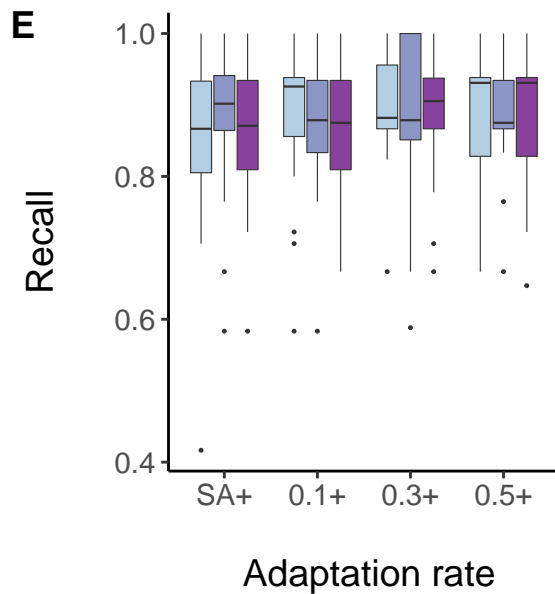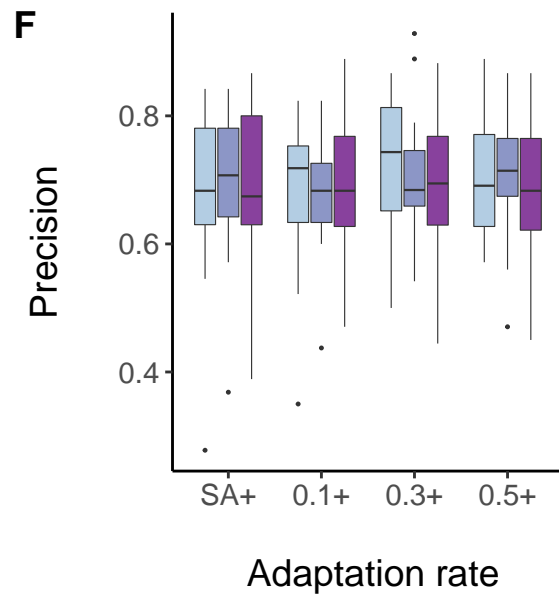

Supplement: S10 Fig — We show box plots corresponding to 20 different transitively reduced DAGs with 16 mutations and for an error rate of 5%. Gray box plots correspond to results of the MCEM algorithm for the true poset. We use the forward sampling scheme with L = 1000 samples. For learning the poset, we fix the ideal acceptance rate to 1/p = 0.0625 and run 25000 simulated annealing iterations. P: true poset, SA: simulated annealing, +: with additional new moves. (PDF) [file pcbi.1008363.s012.pdf]

**A**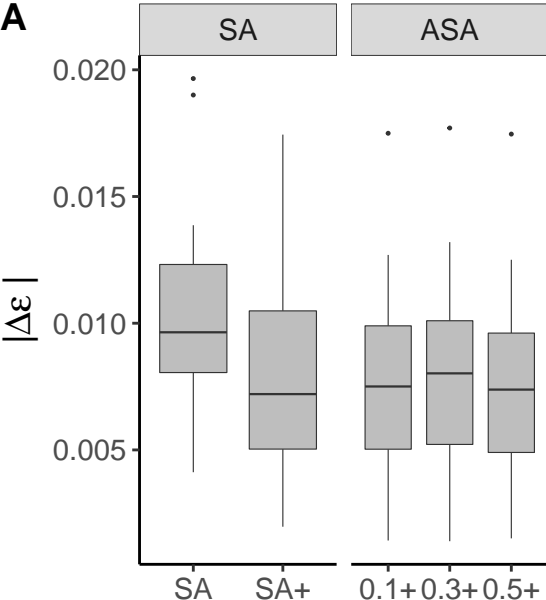**B**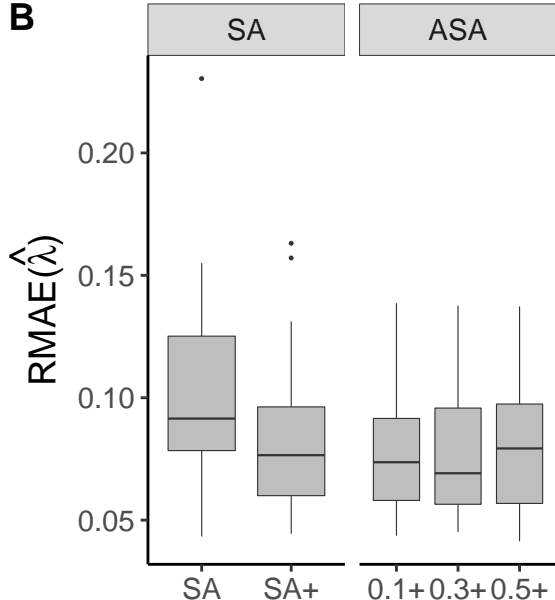**C**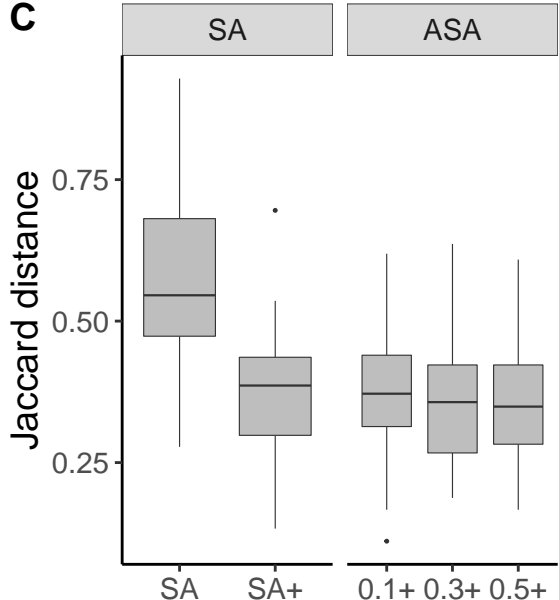

Supplement: S11 Fig — A Absolute error in estimating the error rate parameter ϵ^. B Relative median absolute error (RMAE) of the estimated rate parameters λ^. C Jaccard distance computed on the cover relation sets for the true and estimated poset. We show box plots corresponding to 20 different transitively reduced DAGs for simulated data sets with 16 mutations and an error rate of 5%. We use the forward sampling scheme with L = 1000 samples drawn from the proposal distribution. We fix the ideal acceptance rate to 1/p = 0.0625 and run 25000 iterations of the simulated annealing algorithm. The initial temperature is set to Θ0 = 50 for all runs and for the adaptive simulated annealing three adaptation rates are evaluated (ar = 0.1, 0.3, 0.5). SA: simulated annealing, ASA: adaptive simulated annealing, +: with additional new moves. (PDF) [file pcbi.1008363.s013.pdf]

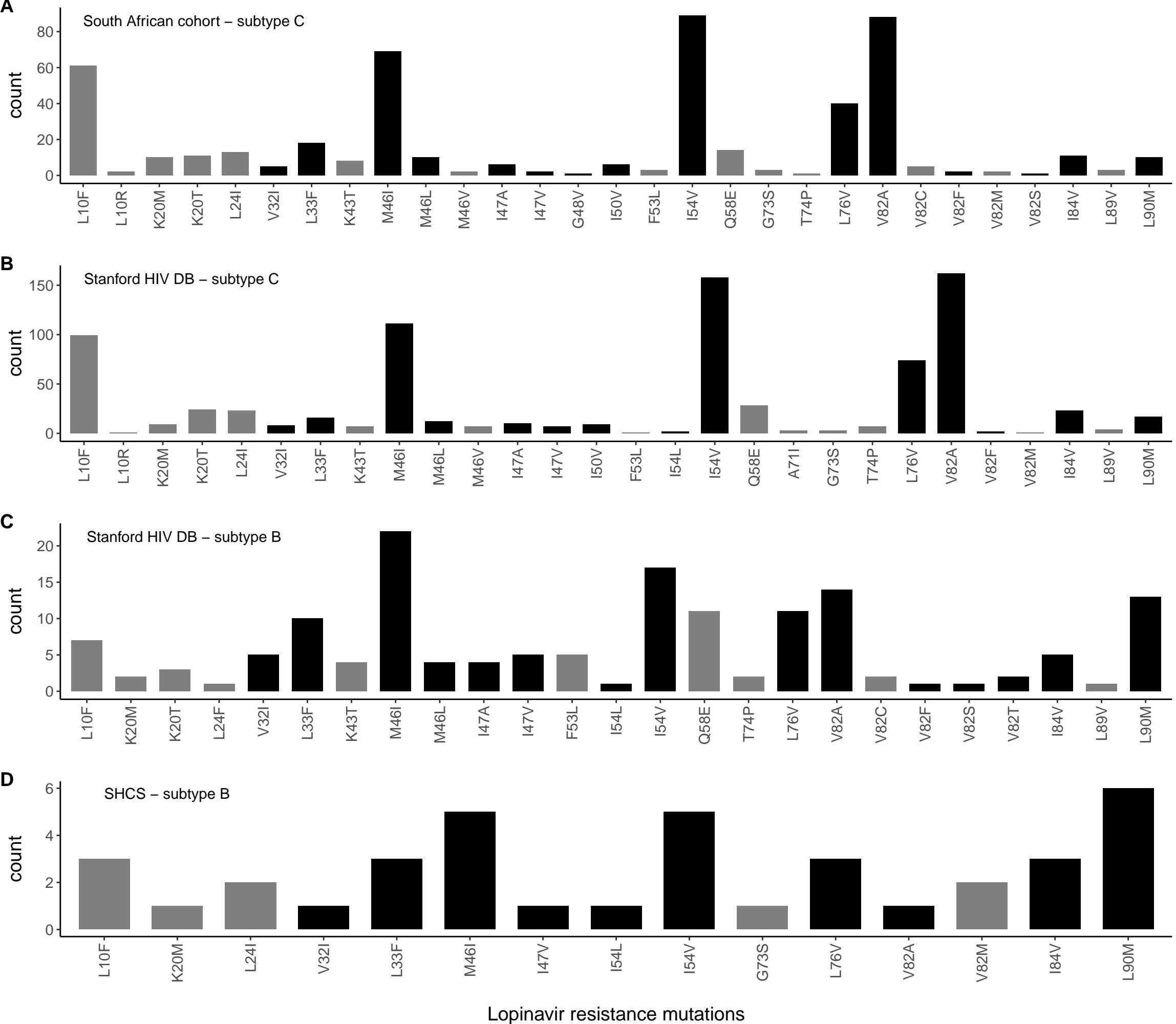

Supplement: S12 Fig — A Data collected from 1064 patients from South Africa (HIV-1 subtype C). B HIV-1 subtype C genotypes retrieved from the HIVDB, excluding genotypes in data set A. C HIV-1 subtype B genotypes retrieved from the HIVDB. D Data obtained from the SHCS corresponding to subtype B genotypes. Major protease inhibitor resistance mutations are shown in black. (PDF) [file pcbi.1008363.s014.pdf]

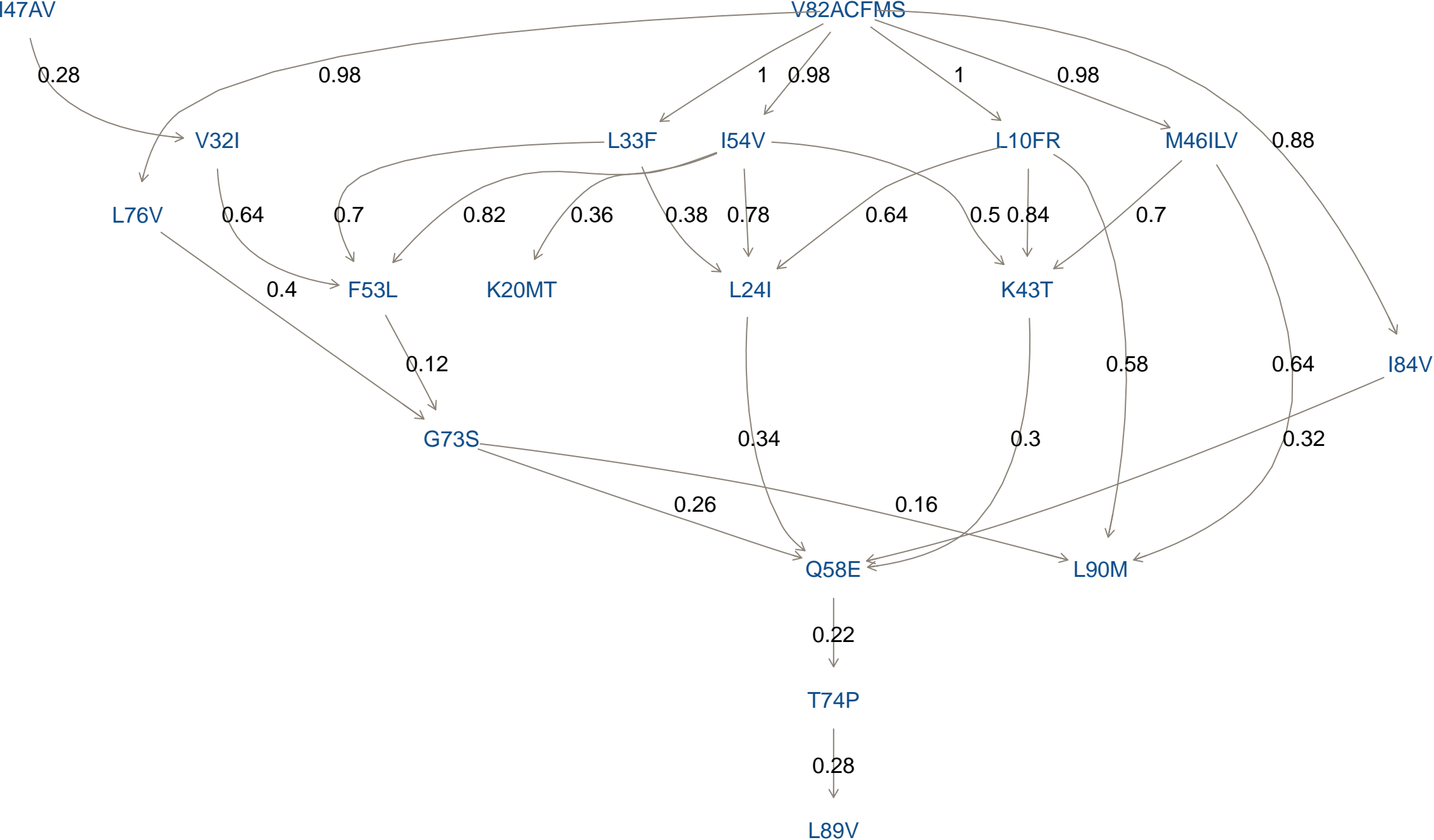

Supplement: S13 Fig — Nodes in the network correspond to amino acid changes in the protease, with mutations at the same locus grouped together. Mutations G48V and I50V are excluded for the comparison of H-CBN2 models, as they are not observed in the subtype B data set. (PDF) [file pcbi.1008363.s015.pdf]

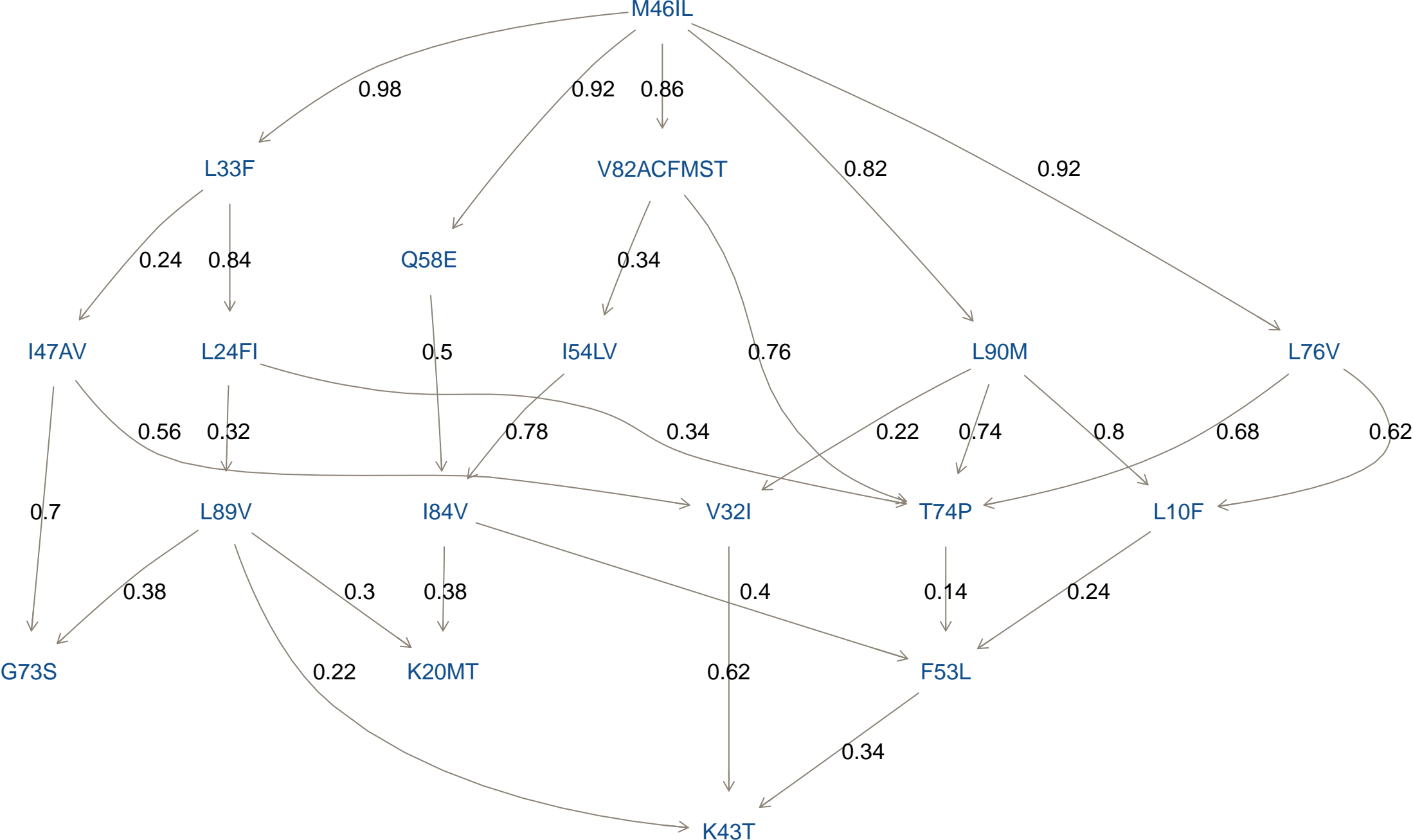

Supplement: S14 Fig — Nodes in the network correspond to amino acid changes in the protease, with mutations at the same locus grouped together. Data sources: the HIVDB and the SHCS. (PDF) [file pcbi.1008363.s016.pdf]
