# Supplementary material for: Comparing mutational pathways to lopinavir resistance in HIV-1 subtypes B versus C
Source: PLoS Comput Biol. 2021 Sep 7;17(9):e1008363. doi: 10.1371/journal.pcbi.1008363 (PMC8448360; doi:10.1371/journal.pcbi.1008363)
Supplement: S9 Fig — We also show the run times of the H-CBN method for posets with up to 14 mutations. The benchmark is conducted on 100 different data sets per poset size, and the number of EM iterations is set to 100. The blue dotted line corresponds to linear scaling, whereas the red line corresponds to quadratic scaling. We conduct the benchmark on two 12-core Intel Xeon E5–2680 v3 processors (2.5 GHz). (PDF) [file pcbi.1008363.s011.pdf]

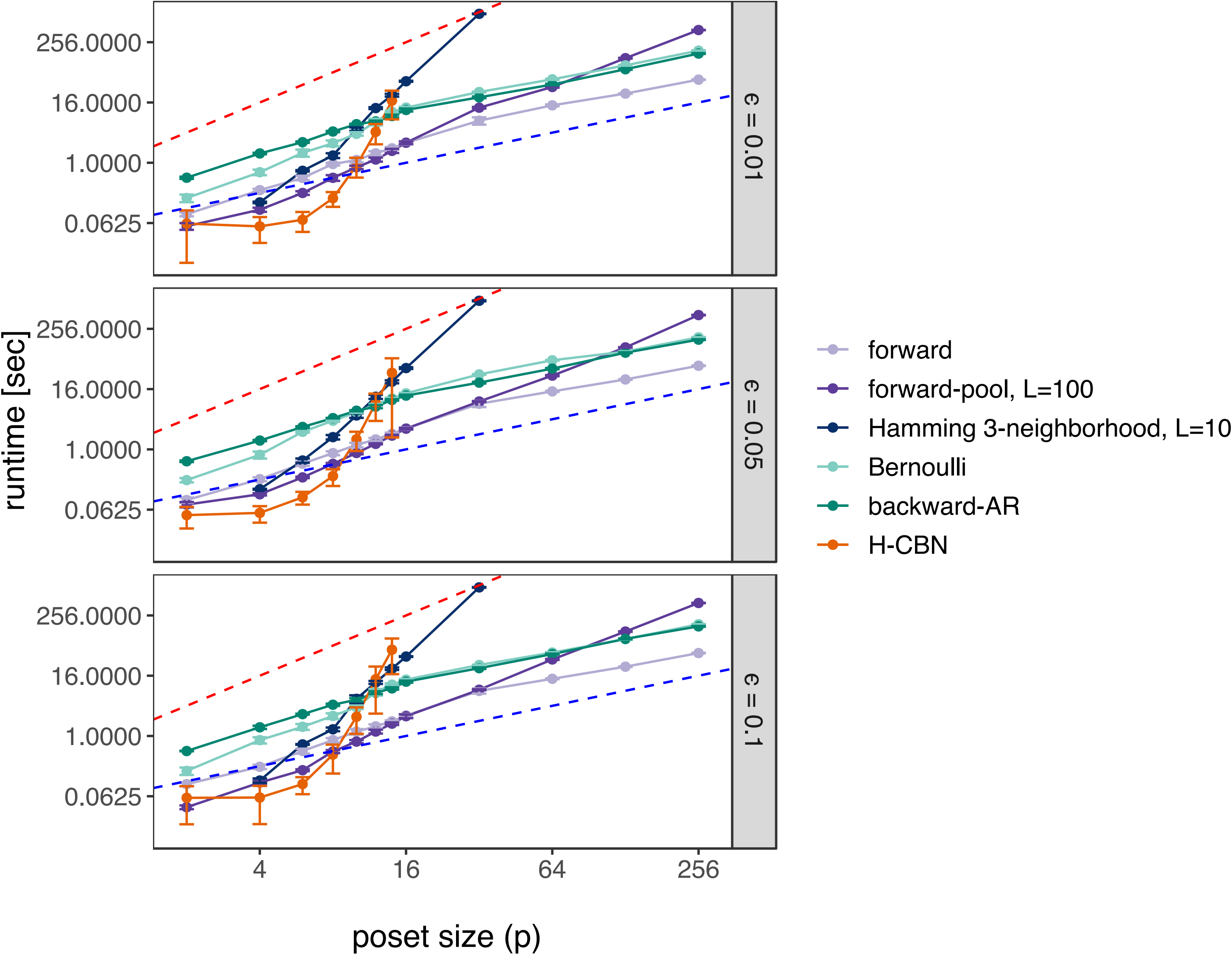

runtime [sec]
256.0000
16.0000
1.0000
0.0625
ϵ = 0.01
ϵ = 0.05
ϵ = 0.1
4
16
64
256
poset size (p)
forward
forward-pool, L=100
Hamming 3-neighborhood, L=10
Bernoulli
backward-AR
H-CBN
