# Supplementary material for: Comparing mutational pathways to lopinavir resistance in HIV-1 subtypes B versus C
Source: PLoS Comput Biol. 2021 Sep 7;17(9):e1008363. doi: 10.1371/journal.pcbi.1008363 (PMC8448360; doi:10.1371/journal.pcbi.1008363)
Supplement: S1 Table — (PDF) [file pcbi.1008363.s017.pdf]

Table S1. Average (range) number of edges in simulated data sets

Number of mutations (p)	Graph density	
	**0.05**	**0.15**
32	24.4 (21-25)	51.5 (41-63)
64	92.5 (83-99)	133.8 (109-165)
128	299.3 (267-333)	302.7 (248-371)
256	760.0 (682-830)	639.8 (582-705)
