# Supplementary material for: Comparing mutational pathways to lopinavir resistance in HIV-1 subtypes B versus C
Source: PLoS Comput Biol. 2021 Sep 7;17(9):e1008363. doi: 10.1371/journal.pcbi.1008363 (PMC8448360; doi:10.1371/journal.pcbi.1008363)
Supplement: S2 Table — The relative error is computed by dividing the absolute error by the absolute value of the true log-likelihood. We note that for the forward-pool sampling, the relative absolute error of the log-likelihood depends almost exclusively on the the size of the initial pool K for L ≥ 10. Similarly, the approximation accuracy of the Hamming k-neighborhood sampling is primarily determined by the extent k of the considered neighborhood. (PDF) [file pcbi.1008363.s018.pdf]

Table S2. Relative error in approximating the log-likelihood via importance sampling. The relative error is computed by dividing the absolute error by the absolute value of the true log-likelihood. We note that for the forward-pool sampling, the relative absolute error of the log-likelihood depends almost exclusively on the the size of the initial pool K for $L \geq 10$. Similarly, the approximation accuracy of the Hamming k-neighborhood sampling is primarily determined by the extent k of the considered neighborhood.

Sampling scheme	Parameters	Relative error
Forward	$L = 10$	0.3909
	$L = 100$	0.0867
	$L = 1000$	0.0134
Forward-pool	$L = 10, K = 200$	0.0530
	$L = 10, K = 800$	0.0177
	$L = 10, K = 1600$	0.0100
	$L = 100, K = 200$	0.0530
	$L = 100, K = 800$	0.0177
	$L = 100, K = 1600$	0.0081
	$L = 10, K = 2000$	0.0095
	$L = 100, K = 2000$	0.0095
	$L = 1000, K = 2000$	0.0095
Hamming 1-neighborhood	$L = 5$	0.0739
	$L = 10$	0.0532
	$L = 50$	0.0332
Hamming 2-neighborhood	$L = 5$	0.0428
	$L = 10$	0.0267
	$L = 50$	0.0111
Hamming 3-neighborhood	$L = 5$	0.0348
	$L = 10$	0.0132
	$L = 50$	0.0050
Bernoulli	$L = 10$	0.2123
	$L = 100$	0.1281
	$L = 100$	0.0262
backward-AR	$L = 10$	0.0896
	$L = 100$	0.0181
	$L = 1000$	0.0133
