# Supplementary material for: Comparing mutational pathways to lopinavir resistance in HIV-1 subtypes B versus C
Source: PLoS Comput Biol. 2021 Sep 7;17(9):e1008363. doi: 10.1371/journal.pcbi.1008363 (PMC8448360; doi:10.1371/journal.pcbi.1008363)
Supplement: Attachment — Submitted filename: Response_to_reviewers_H-CBN2.pdf [file pcbi.1008363.s022.pdf]

Response to Reviewers

PCOMPBIOL-D-20-01701R1

Comments to the Authors:

Please note here if the review is uploaded as an attachment.

Reviewer #1: In the article, the authors present an extension of the Hidden Conjunctive Bayesian Network framework (H-CBN) for the inference of mutational graphs from cross sectional data (previously developed by some of the authors), aimed at improving the overall scalability, by proposing different sampling schemes and a new adaptive simulating annealing strategy.

They apply their method (named H-CBN2) to infer the mutational/evolutionary pathways related to lopinavir resistance in HIV-1 subtypes B and C and they compare the models, finding significant differences.

As a first general comment, the article is technically sound, even if the presentation of goals and assumptions is not always clear and effective.

On the one hand, the core of the article is the presentation of an extension of an already existing computational approach, aimed at addressing scalability issues (most of the main text and the whole SM), and this would plainly position the article within the Methods category.

On the other hand, starting from the title, the abstract and the author summary, the declared goal and focus of the manuscript seem to be exquisitely application/translational.

I would strongly suggest to choose either a methodological or a translational "cut" and to revise the overall organization accordingly and in a consistent fashion, starting from the title, abstract, summary, proportion of text in the sections, etc.

Thank you for this comment! We believe that both aspects are important for this manuscript. Indeed, we emphasize in the summary that the method development was largely driven by the particular application. The choice of the model, namely H-CBN, is now better motivated in the introduction. First, the H-CBN model typically outperforms competitors (we added references to previous simulation studies) and, second, the model incorporates a superior error model compared to other CT-CBN models. To improve the scalability of the model, we incorporate a novel approximate inference scheme for parameter estimation, which allows us to consider networks with about 20 mutations. This extension was necessary in order to perform meaningful comparisons between mutational pathways to lopinavir resistance in

HIV-1. We provide a more succinct description of the H-CBN model in the methods section, and for more details, we refer the reader to the supporting information or original publication.

From the methodological perspective, a more comprehensive comparison against competing methods is surely missing, since the authors only compare their results with the model of H-CBN, which is also the inspiration/source of their approach.

There is plenty of methods to reconstruct mutational trees/graphs (which the authors themselves cite) and at least some of the most recent/used should be included in the comparison.

Many of the cited models provide insights into statistical dependencies between resistance mutations, but do not explicitly model the temporal ordering among such mutations, for example, Bayesian networks (Deforche et al., 2006; Deforche et al., 2008; Marie and Gordon, 2019) and the Cox proportional-hazards model (Lawyer et al., 2011). Models that describe the accumulation of mutations over time include Markov processes (Foulkes and De Gruttola, 2003; Buendia et al., 2009; Healy and DeGruttola, 2006), mutagenetic trees and tree mixtures (Desper et al., 1999; Beerenwinkel et al., 2005), Conjunctive Bayesian networks (Beerenwinkel et al., 2007; Beerenwinkel and Sullivant, 2009; Gerstung et al., 2009) and CAPRI (Ramazzoti et al., 2015). However, this study was motivated based on the availability of a large data set of HIV-1 subtype C genotypes from South African individuals treated with lopinavir. The treatment history is not available, nor the measurement times. Therefore, using models that require longitudinal observations, such as Foulkes and De Gruttola (2003) and Healy and DeGruttola (2006), is not possible for our application.

Comparison of the CBN model, including H-CBN, against related models show that CBNs oftentimes perform better (Diaz-Uriarte, 2017; Diaz-Uriarte and Vasallo, 2019).

Following the reviewer's suggestion, we have included a comparison of H-CBN2 to the MC-CBN method which uses a mixture error model. We find that for noisy genotypes, H-CBN2 outperforms MC-CBN in terms of the F1 score for the reconstructed network edges. For error-free data, both methods perform equally well (Figure 2F-G). For a more comprehensive comparison of the CBN/H-CBN model against related methods, we included references in the text to previous studies (Hainke et al., 2012; Diaz-Uriarte, 2017; Diaz-Uriarte and Vasallo, 2019).

Furthermore, a comparison with phylodynamics approaches is missing, see, e.g. [Kühnert, D., Kouyos, R., Shirreff, G., Pečerska, J., Scherrer, A. U., Böni, J., ... & Stadler, T. (2018). Quantifying the fitness cost of HIV-1 drug resistance mutations through phylodynamics. PLoS pathogens, 14(2), e1006895]. This article is not even cited, despite being applied to one of the datasets employed in this work.

How do the proposed approach compares against standard phylodynamic approaches?

Although both our work and that presented by Kühnert *et al.* study drug-resistance mutations in HIV using data from the Swiss HIV Cohort Study, each addresses quite different research

questions. Kühnert *et al.* are interested in the transmission fitness of viral lineages between hosts, whereas we investigate the within-host evolution of drug-resistance under a given drug-selective pressure. Moreover, Kühnert *et al.* employed sequences from treatment-naïve patients (as opposed to sequences after drug exposure) and reported results, such as phylogenies and fitness parameters, separately for each individual drug resistance mutation. Their approach does not model dependencies between drug resistance mutations at all, which is the focus of our manuscript. Thus, the scope of their study is entirely different from ours.

More generally, phylogenetic and phylodynamic methods address related, yet different, research questions, compared to progression models. In standard phylogenetic analyses the goal is to learn the evolutionary relationship among the set of observed sequences. By contrast, progression models are used to learn the order constraints between mutations in a way that allows generalization to previously unseen mutation patterns. For instance, progression models are used for interrogating, for a given viral population (characterized by its consensus sequence / mutation pattern), which mutations are likely to emerge next under a certain drug pressure. See also the answer to the next question.

Would not it be more sound to infer a phylogeny of samples instead than a mutational graph, attaching the mutations to the phylogenetic model a posteriori? The authors should address this important concern.

The two model classes serve different purposes, make different assumptions, and are applied in different situations: Phylogenetic models assume a joint evolutionary history of all samples and aim at reconstructing it, and they assume independence among loci. Progression models, on the other hand, assume independence of the samples (i.e., no joint evolutionary history) and aim at inferring dependencies among mutations at different loci. Progression models are applicable if sequences have evolved independently (here in different hosts) under similar constraints (here the same selective drug pressure). We note that the assumption of independent evolution of drug resistance in each host ignores transmission of drug resistant viruses. This simplification is justified by the low rates of drug resistance transmission owing to viral suppression (and hence reduced transmissibility) under treatment (Baxter *et al*, HIV Med., 2015, 10.1111/hiv.12236). We agree with the reviewer that phylogenetic methods are used for modeling viral sequences derived from the same patient (i.e., when they do share an evolutionary history), including in the context of HIV drug resistance (Buendia et al., 2009), but this is not the setting of the present manuscript. The relation of our approach to phylogenetic methods is now mentioned in the Discussion.

Other tree models have been adopted to progression models and used to derive dependencies among mutations (Desper *et al.*, 1999; Desper *et al.*, 2000; Beerenwinkel *et al.*, 2005). However, the tree-like dependency structure among mutations inferred by these methods is not due to inheritance (as in phylogenetic trees), but due to the combined effect (i.e., epistatic interactions) of mutations on the phenotype (e.g., drug resistance). However, these interactions are not necessarily tree-like and there is no a priori reason to assume a tree model for their inference other than maybe computational feasibility. Indeed, tree models are more restrictive than CBNs (e.g., they cannot model the dependency of a mutation on more than

one other mutation -- converging edges in the graph), and consequently CBNs have been shown to outperform tree models when the true dependency structure is not tree-like (Hainke *et al.*, 2012). These explanations have also been added to the Introduction.

An further general methodological concern regards the effectiveness of population models inferred from cross-sectional data, especially in presence of high or unknown levels of genomic heterogeneity, as typically observed in cancer evolution scenarios, which is the field within which H-CBNs were originally developed.

In this regard, in a recent work some of the authors introduced the concept of predictability, which roughly measures the repeatability of the evolutionary patterns of a given mutational graph [Hosseini, S. R., Diaz-Uriarte, R., Markowetz, F., & Beerenwinkel, N. (2019). Estimating the predictability of cancer evolution. Bioinformatics, 35(14), i389-i397].

This aspect must be evaluated if one aims at delivering experimental hypotheses with translational relevance and could help in casting a light on the intrinsic heterogeneity of the generative process underlying resistance in HIV.

Hosseini et al. (2019) proposed a notion of predictability which is based on the normalized entropy of the probability distribution of mutational pathways. In particular, probabilities of mutational pathways were derived by using the H-CBN model. We estimated the predictability in our data sets using the H-CBN model (see table below) and 5 bootstrap samples. For each bootstrap sample, we use the approximation for large numbers of mutations by averaging over all subsets with size $n'=4$. We find that the predictability is generally high (and substantially higher than zero), suggesting that evolution of drug resistance under selective pressure of lopinavir is reproducible.

**Dataset**	**Predictability (mean, [range])**
South African cohort	0.73 [0.71, 0.75]
Subtype B	0.74 [0.68, 0.77]
Subtype C (non South African)	0.69 [0.68, 0.70]

The high evolutionary predictability is consistent with the consensus posets reported in Fig. 3 and 4, which contain a large number of stable dependencies (bootstrap support > 0.7). Thus, the development of resistance to lopinavir is an evolutionary process which is reproducible in independent hosts and hence predictable to a large extent. The main goal of the present paper is to detect differences in (the reproducible part of) this process between HIV-1 subtypes B versus C.

Moreover, the suitability of conjunctive models has longly been questioned, for instance with respect to mutual exclusivity events (e.g., negative epistasis) or loss of mutations.

These aspect should be considered, particularly when considering a significantly high number of genomic events/mutations, which is the scenario that the authors aim to address by improving the scalability w.r.t. standard H-CBNs.

In general, CBN models do not incorporate fitness nor any other phenotype, and hence epistasis cannot be modeled explicitly, but its consequences (in terms of feasible genotypes and mutational pathways and their timing or probability) may or may not be representable. From the poset, one can obtain the set of feasible genotypes which are compatible with the order constraints defined by the poset. The set of all genotypes compatible with the poset is the genotype lattice, which represents all possible paths from the starting genotype (often the wild type) to a fully-mutated type. It follows that genotypes that are not compatible with the poset have probability zero (albeit observable due to the error process) and are not part of the genotype lattice. To make a connection between CBN models and the fitness landscape, it is usually assumed that mutations only improve fitness (i.e., accumulation of advantageous mutations; here drug resistance mutations) and evolution goes in the direction towards increasing fitness (e.g., no back-mutations). As reported by Diaz-Uriarte, CBNs can represent (the effects of) fitness landscapes with sign epistasis (including some forms of negative epistasis), but not reciprocal sign epistasis (Diaz-Uriarte, Bioinformatics, 2013, 10.1093/bioinformatics/btx663; Diaz-Uriarte and Vasallo, PLoS Comput. Biol., 2019, 10.1371/journal.pcbi.1007246). It is also worth noting that if one were to relax the conjunctive constraint of CBNs and thus allow representing more complex interactions, the number of parameters would increase exponentially with the number of mutations which will be prohibitive for our application. This is the case for general Bayesian networks which do not explicitly model the partial temporal ordering among mutations and hence can not be readily interpreted as progression models, nor used for predicting the occurrence of mutations (Deforche et al., 2006; Deforche et al., 2008; Marie and Gordon, 2019). We have now mentioned this limitation of the model in the Discussion.

Despite limitations in the expressiveness of mutational pathways, CBN models have been employed successfully in various applications, such as, for example, the estimation of an individualized genetic barrier assuming non-decreasing resistance phenotypes (Beerenwinkel et al., PLoS Comput. Biol, 2013, 10.1371/journal.pcbi.1003203) and predictability of evolution under the strong selection weak mutation assumption (Hosseini et al., Bioinformatics, 2010, 10.1093/bioinformatics/btz332). For the present application of network comparison, we do not directly compare the genotype lattices, but instead we compare the posets (or Hasse diagrams) representing the mutational order constraints.

Besides, in [PLoS One. 2010 Jul 7;5(7):e11345. doi: 10.1371/journal.pone.0011345.] back mutations are indeed observed and "resistant virus disappeared rapidly after treatment interruption and was undetectable as early as after 3 months".

Yes, CBN models as other approaches to model accumulation of mutations incorporate the assumption of no back-mutations being allowed. The assumption is often justified, because each mutation provides a selective advantage (e.g., drug resistance mutations under continuous treatment) and hence its disappearance is unlikely. In our application, we are

interested in development of lopinavir resistance and there are no treatment interruptions/changes in our data sets. Since mutation loss under uninterrupted treatment is rather rare and even upon discontinuation of treatment (Zhang *et al.,* PLoS genetics, 2020), we argue that this effect is negligible.

How do the authors can assess the robustness of their results, which seem to be highly dependent on sampling/sequencing timing?

If the sampling times are unknown, they are assumed to be exponentially distributed random variables (Beerenwinkel and Sullivant, Biometrika, 2009; Gertung *et al.*, Bioinformatics, 2009). The exponential distribution is perhaps the simplest continuous distribution which has support on the non-negative real numbers and is a common choice to describe time between events (i.e, independent Poisson processes). Nonetheless, when the sampling times are known, these can be incorporated into the model (OT-CBN model: Montazeri *et al.*, Biostatistics, 2015, 10.1093/biostatistics/kxv019) resulting in inferred models with higher likelihoods compared to the models in which sampling times are marginalized out.

In a general sense, the robustness of the network estimation is addressed by using bootstrapping, and to compare mutational networks we use a permutation test to account for any such uncertainties.

In addition, how do the proposed approach accounts for positive epistasis, which is significantly present in HIV evolution (see, e.g., Bonhoeffer, Sebastian, et al. "Evidence for positive epistasis in HIV-1." Science 306.5701 (2004): 1547-1550.9)?

While CBN models do not incorporate a notion of fitness, some forms of positive epistasis can be modeled as the gain of mutation B after mutation A (A → B) with high transition probability, i.e., P(B=1 | A=1) large. We reiterate here that our model does not account for fitness values, but it models dependencies among mutations which are assumed to be the result of mutation and selection (and possibly other evolutionary forces). The types of dependencies that can be modeled are restricted. The CBN is more general than tree models (see also answer above) and it includes positive and negative epistatic interactions, but it is still far away from covering all possible interactions. In fact, the genotype probabilities are parameterized by n parameters, one for each mutation, but the number of genotypes can be up to 2^n. This model choice reflects our tradeoff between goodness-of-fit and model complexity in order to achieve good generalizability with a computationally feasible model.

For instance, in [Zhang, Tian-hao, et al. "Predominance of positive epistasis among drug resistance-associated mutations in HIV-1 protease." PLoS genetics 16.10 (2020): e1009009] the authors claim: "We found that although resistance associated mutations greatly reduce replication fitness, they interact positively to alleviate the mutational load. These genetic interactions, termed epistasis, increase the ruggedness along the evolution paths, restricting resistance associated mutations from reversal."

Zhang *et al.* found that reverting to wild type is unlikely due to positive epistasis resulting in fitness valleys, hence supporting the CBN assumption of not allowing back mutations (loss of mutations). Furthermore, some forms of positive epistasis can be modelled by CBNs as explained in the previous answer.

In the same paper, the authors find at least two mutations (L10F and M46) with positive epistasis, which is in apparent contrast to the model presented in Figure 6 of the current manuscript, in which such mutations are located in independent branches.

The Zhang *et al.* work was not conducted in the context of any particular drug pressure nor subtypes, therefore their findings are not directly comparable to ours. As explained above, the mutational pathways we infer reflect properties of the underlying fitness landscape, and the fitness landscape is affected massively by drug treatment.

From the application perspective, the presentation of the translational results derived from the models is quite poor and misses many important relevant aspects in HIV resistance analysis. More detail follows.

First, the article is completely missing the key issue of intra-host genomic variability.

For instance, the concept of quasispecies is not even mentioned and this is serious flaw, considering that the declared goal is to investigate resistance in viral evolution (see, for instance, the many thorough works by Esteban Domingo).

Genotype resistance testing based on Sanger sequencing is still the gold standard and major source of information on drug resistance development in publicly available databases such as the Stanford HIV Drug Resistance Database. Since only abundant variants in the virus population can be identified with Sanger sequencing, no intra-host variability can be addressed using these data. CBN models are designed for such Sanger sequencing data which does not capture intra-host genetic variation.

A basic premise for the CBN model is that a mutation that allows the virus to reproduce despite treatment confers a selective advantage and becomes dominant in the population. Then, drug-resistance develops by the subsequent expansion of viral types harbouring multiple mutations in a stepwise fashion. The focus in using this modelling approach is not to understand in detail within-host viral evolution, but rather to determine shared constraints in the development of drug resistance from patients treated with the same drug/regime.

Quasispecies genetic "swarms" are supposed to underlie most of the adaptive potential of viruses to the immune system response or to antiviral therapies, also for HIV [see, e.g., Najera, Isabel, et al. "Pol gene quasispecies of human immunodeficiency virus: mutations associated with drug resistance in virus from patients undergoing no drug therapy." Journal of virology 69.1 (1995): 23-31.; Boutwell, C. L. et al. Todd M. Viral Evolution and Escape during Acute HIV‑1 Infection. The Journal of Infectious Diseases 202, S309–S314, https://doi.org/10.1086/655653 (2010). Hedskog, Charlotte, et al. "Dynamics of HIV-1 quasispecies during antiviral treatment dissected using ultra-deep pyrosequencing." PloS one

5.7 (2010): e11345. Vandenhende, M. A. et al. Prevalence and evolution of low frequency HIV drug resistance mutations detected by ultra deep sequencing in patients experiencing first line antiretroviral therapy failure. Plos One 9, e86771 (2014), Da Wei Huang, Castle Raley, et al. "Towards better precision medicine: PacBio single-molecule long reads resolve the interpretation of HIV drug resistant mutation profiles at explicit quasispecies (haplotype) level." Journal of data mining in genomics & proteomics 7.1 (2016).]

It is clear that the resolution of consensus sequences does not allow to evaluate the presence of low frequency variants or to deconvolve the quasispecies composition (as the author admit in the Discussion), yet this issue might significantly impact the overall translational relevance of this work and should be explicitly addressed.

Low-frequency variants may play a role in the evolution of drug-resistance, but only consensus sequences are readily available to us, hence low-frequency mutations cannot be addressed in the framework of this study.

Moreover, the authors state "However, when viral replication is inadequately suppressed, the virus may acquire mutations 6 which confer resistance to cART,".

Despite being a complex topic, it was hypothesized that treatment failure might be instead likely caused by the preexistence of resistant mutants (see, e.g., Proc Natl Acad Sci U S A. 2000 Jul 5; 97(14).

We agree that this may be the case. However, we argue that it will not make a difference to the model and its applicability, but a slight difference in interpretation. Generally, the CBN model describes the accumulation of mutations at the consensus level. Hence, the rates associated with each mutation (sometimes called "rates of evolution") generally account for all underlying processes, such as selection of pre-existent or generation and selection of novel mutations. In particular, if a mutation was pre-existing as a low-frequency variant at the start of treatment, the corresponding rate does not describe both emergence and fixation at the population level, but only the latter.

How can the H-CBN2 approach be effective in addressing the existence of pre-existing mutations, for instance if present in a subset of samples of the cohort?

It can not, and it is not the subject of our manuscript. We have addressed this question from the perspective of low-abundance mutations in the preceding question. For mutations that are present at the population level, including rare cases of transmitted resistance, one can simply redefine the wild type (or starting genotype) per sample/patient if genotypes at start of treatment were available. However, these data are not available to us. More relevant to our application is that the South African Antiretroviral guidelines only include lopinavir alone or boosted with ritonavir as protease inhibitors (PI), and also the genotypes obtained from the SHCS correspond to patients treated with lopinavir as the first PI. Since we are only considering accumulation of mutations on the protease, it is justified to neglect contributions from previous treatments.

The authors also claim: "A better understanding of the underlying evolutionary process leading to resistance is believed to be crucial for predicting therapy outcome and designing effective therapy sequences ".

I might have missed some detail, but if the therapy underlies the emergence of resistance-related mutations, the model cannot be used for predictions, but only for a posteriori explanations/descriptions.

The model allows for predicting which mutations will accumulate in which order and hence allows for estimating the probability of drug resistance development. For instance, Beerenwinkel *et al.* (PLoS Comput. Biol, 2013, 10.1371/journal.pcbi.1003203) proposed a framework to quantify the individualized genetic barrier or, in an opposite manner, the potential of a virus population to develop resistance to a certain drug. This approach incorporates the CBN model to compute the transition probabilities between genotypes and is used to predict clinical outcomes. More generally, combining models of accumulating mutations with indicators of treatment success/failure (e.g., viral load) and drug resistance phenotypic information allows to model treatment outcome, which is relevant for therapy optimization (see e.g., Beerenwinkel *et al.*, Bioinformatics, 2005, 10.1093/bioinformatics/bti654; Rahnenführer *et al.*, Bioinformatics, 2005, 10.1093/bioinformatics/bti312; Altmann *et al.*, Antivir Ther., 2007; Rosen-Zvi *et al.*, Bioinformatics, 2008, 10.1093/bioinformatics/btn141; Parbhoo *et al.*, AMIA Jt Summits Transl Sci Proc. 2017).

Minor

Some sentences are involuted.

For instance, in the abstract "the maximum likelihood mutational networks for subtypes B and C share only 7 edges (Jaccard distance 0.802) and imply many different evolutionary pathways.

What is an edge? How many edges are present in the models? Why reporting the Jaccard distance?

We use the Jaccard distance for comparing the cover relation sets, but we agree that it might only become clear after defining the poset. We replaced this sentence with the following: "The inferred mutational networks are found to be significantly different sharing only a sub-network with 5 edges (i.e., constraints on the order of accumulation of mutations) and mutation at residue 54 as the parental event".

How were the error rates of the simulated datasets chosen?

The error rates chosen for simulation were based on previous simulation studies (Gerstung et al., 2009; Montazeri et al., 2016) and by considering our particular application. The data analyzed here is obtained from HIV drug resistance testing, where possible sources of technical noise include reverse transcription, PCR amplification, and sequencing. The reported error rates for Sanger sequencing vary from 10^{-5} to more than 0.01 (Hoff, 2009). Taken together, we have therefore chosen to test the accuracy of the approximate inference algorithm with $\varepsilon = 0.01,\ 0.05$ and 0.1, which are more challenging cases for the parameter inference. For the comparative assessment of the structure learning, we also simulated data with smaller error rates ($\varepsilon = 0,\ 0.001$and 0.05), including the limit case of error-free genotypes to assess the advantage of the latent error model compared to the more simple mixture error model even under these milder conditions. We have now motivated the choice of the error rates in the Methods section.

abstract: Antiretoviral --> Antiretroviral

Thanks, it has been changed.

Fig 1A is a very minor modification of Fig 1B of the article "Quantifying cancer progression with conjunctive Bayesian networks" and which should be mentioned.

While making the methods section more succinct, we decided to exclude the figure and refer the reader to the original publications.

Reviewer #2: The paper describes a general method for inference of mutation accumulation rates and orders of their appearance from genomic data. The authors propose a maximal likelihood-based graphical model for inference of evolutionary parameters of interest. The most general version of the model consists of exponential number of terms and variables and thus is computationally intractable; therefore, the paper proposes a mixed approach combining Monte Carlo Sampling and Expectation Maximization likelihood optimization for numerical parameter estimation and simulated annealing-based local search for graphical model inference. The proposed method is applied to the analysis of the emergence of lopinavir resistance mutations in HIV.

Overall, the proposed general modelling framework is elegant and may be of interest to the computational genomics community. However, I have some concerns about presentation, proposed approach and its validation.

1) The title is somewhat misleading. Judging by the way the manuscript is written, its major subject is the methodology rather than applications. The method is rather general and has nothing specifically related to viral drug and therapy resistance. As a matter of fact, it seems like it can be applied to any kind of viral or cancer genomic data.

Thank you for this comment! Our work focuses on viral genomics, however, the H-CBN model can indeed be used for modelling any accumulation of genetic alterations, and the comparison of progression models can also be used to compare any two groups, e.g., viral subtypes or cancer types, which is now highlighted in the abstract, summary and discussion. Following this suggestion, we have modified the abstract to reflect better the development of methods allowing the comparison of mutational networks.

2) Abstract claims that the proposed method can handle a large number of resistance mutations. However, the validation results on simulated data does not support this claim. Possibly it is meant that the method still can handle more mutations than its competitors – in that case it should be emphasized.

Yes, we apologize for being imprecise. We have developed H-CBN2 to address the scalability shortcomings of its predecessor (H-CBN), but the strongest motivation was to be able to provide an answer to the question: does evolution of drug resistance in HIV-1 subtype C differ from subtype B? This question is important, because subtype C is highly abundant in sub-Saharan Africa where lopinavir is now being used, yet most of our current knowledge on lopinavir resistance development is derived from subtype B genotypes. We performed most of the hyper-parameter tuning with this application in mind. We agree that with the current settings (e.g., number of samples drawn from the proposal distribution), we can only assert that H-CBN2 can handle a larger number of mutations, and we changed the corresponding sentence in the abstract accordingly.

3) The motivation part should be improved. Specifically, it is claimed that the major novelty of the study is the comparison of mutational pathways between different HIV subtypes. However, it is not entirely clear, why such comparison cannot be made using existing methods, and why development of a new method facilitates such comparison.

While the statistical test could, in principle, be used to compare networks inferred using other methods, we choose to use the H-CBN model for two main reasons: first, the H-CBN model typically performs better compared to other models in benchmarking studies (Diaz-Uriarte, 2017; Diaz-Uriarte and Vasallo, 2019) and, second, the H-CBN offers an improved error model compared to other CT-CBN models. To be able to include more than 14 mutations in the application, we implemented the approximate inference scheme for parameter estimation of the H-CBN model. Both points are now made more explicitly in the introduction.

4) The relation between the proposed approach and standard phylogenetic methods is mentioned only in passing. However, I feel that this relation needs to be discussed in greater detail. It is unclear why mutational pathways and rates cannot be inferred using classical maximum likelihood or Bayesian phylodynamics methods with relaxed molecular clock and ancestral states reconstruction.

The two model classes serve different purposes, make different assumptions, and are applied in different situations: Phylogenetic models assume a joint evolutionary history of all samples and aim at reconstructing it, and they assume independence among loci. Progression models,

on the other hand, assume independence of the samples (i.e., no joint evolutionary history) and aim at inferring dependencies among mutations at different loci. Progression models are applicable if sequences have evolved independently (here in different hosts) under similar constraints (here the same selective drug pressure). We note that the assumption of independent evolution of drug resistance in each host ignores transmission of drug resistant viruses. This simplification is justified by the low rates of drug resistance transmission owing to viral suppression (and hence reduced transmissibility) under treatment (Baxter *et al*, HIV Med.*,* 2015, 10.1111/hiv.12236). We agree with the reviewer that phylogenetic methods are used for modeling viral sequences derived from the same patient (i.e., when they do share an evolutionary history), including in the context of HIV drug resistance (Buendia et al., 2009), but this is not the setting of the present manuscript. The relation of our approach to phylogenetic methods is now mentioned in the Discussion.

Other tree models have been adopted to progression models and used to derive dependencies among mutations (Desper *et al*., 1999; Desper *et al.*, 2000; Beerenwinkel *et al.*, 2005). However, the tree-like dependency structure among mutations inferred by these methods is not due to inheritance (as in phylogenetic trees), but due to the combined effect (i.e., epistatic interactions) of mutations on the phenotype (e.g., drug resistance). However, these interactions are not necessarily tree-like and there is no a priori reason to assume a tree model for their inference other than maybe computational feasibility. Indeed, tree models are more restrictive than CBNs (e.g., they cannot model the dependency of a mutation on more than one other mutation -- converging edges in the graph), and consequently CBNs have been shown to outperform tree models when the true dependency structure is not tree-like (Hainke *et al*., 2012). These explanations have also been added to the Introduction.

5) The error model used in the study seems to be too simplistic. The developed method is applied to genotypes produced by different sequencing experiments, sequencing platforms, and laboratories. Therefore, the assumption of the same error rate for all genotypes is unrealistic.

While the error model used in this work is an improvement over a more simple mixture model (see Introduction), we share these concerns as mentioned in the discussion. However, there is always a trade-off between faithfulness and tractability of the model. As seen in the simulation study, it is already challenging to perform inference using the current error model with the number of genotypes which are typically available. Furthermore, for the datasets considered in this work, using a more complex error model is likely to lead to overparameterized models and the necessary detailed information, such as sequencing platform and laboratories, are not generally available.

In addition, we used consensus sequences obtained by Sanger sequencing, a fairly standardized method which is often taken as it is in many applications. This is because Sanger sequencing error rates are typically small and, in fact, the inferred error rates for the datasets considered in this work range from 0.4% to 1%. Therefore, it is reasonable to assume that these confounders are unlikely to play a major role. It is nevertheless crucial to model errors, as the poset and lattice structure can otherwise be affected strongly.

6) The validation on simulated data is quite limited. The method is basically compared with itself under different settings. It would be desirable to add comparisons with existing methods or at least with some alternative approaches.

Following this suggestion, we compare the H-CBN2 against the MC-CBN method which implements a mixture error model (Figure 2F-G). For a more comprehensive comparison of the CBN/H-CBN model against related methods, we include references to previous studies (Hainke et al., 2012; Diaz-Uriarte, 2017; Diaz-Uriarte and Vasallo, 2019). Our focus here is on scaling this method to the application we address.

7) In simulation study results, it is unclear why the absolute error is reported for genotype error rate and the relative error – for mutation rate. I believe the same relative error should be reported for all parameters.

Thanks! It has been changed (Fig. 2 and Supplementary Fig. 7).

8) It is unclear why the results for simulated annealing algorithm are reported only for 16 mutations. If the results for larger datasets are poor (as Discussion seems to confirm), they still need to be reported. If this is the case, then is it related to optimality of scalability problems?

We scanned the hyper-parameter space for a problem size that is relevant for our application. Nonetheless, we have now included a comparison of the HCBN-2 method to MC-CBN method for two sizes: 16 and 24 mutations. We find that for noisy genotypes, H-CBN2 outperforms MC-CBN in terms of the F1 score for the reconstructed network edges, and for error-free data, both methods perform equally well (Figure 2F-G).

9) Judging by Fig. 3, B, it seems like the algorithm consistently underestimates mutation rates for larger numbers of mutations. Can it be explained? If such biases are possible, how they will affect the statistical comparison of CBN models?

Thank you very much for pointing this out. Using our simulation settings, in particular the number of observations (N) and (average) number of edges in the true underlying network, the chance of observing a given mutation which depends on the occurrence of many predecessors decreases as the poset size increases. Many of such mutations are only effectively encountered in the simulated datasets as a consequence of the noise. Therefore, there is no evidence in the data to estimate the corresponding rate parameters (lambdas).

In order to reduce these biases, a natural choice is to increase the number of observations or to remove mutations from the model that are rare. Alternatively, we consider scenarios with more sparse networks, which generally results in fewer low-probability genotypes. While reducing the number of constraints (or the number of edges in the network), the fraction of independent mutations increases and it is more likely to observe such mutations for chosen simulation settings. We have added an additional figure to the supplementary material to illustrate this point (Supplementary figure S8).

We note that in order to account for these uncertainties that finite-size data sets represent for learning the network, we resort to a permutation test.

10) How statistically significant is the fact that the subtype-specific H-CBN2 posets share some relations?

Observing similarities between mutational networks under the same drug pressure, even for different HIV subtypes, is not considered surprising. Indeed, the null hypothesis of our test states that there are (in the absence of noise) no differences. However, since the mutational networks are learned from inherently noisy (finite) observational data, we can not expect to reconstruct identical networks. To be able to address the question of whether and if so how significantly different inferred posets are, while accounting for uncertainties of poset inference, we propose the customized permutation test described in the Methods section. Briefly, we use the Jaccard distance between two posets to quantify the dissimilarity between them and ask how surprising it is to observe at least this distance for data sets coming from the same underlying poset (which is our null hypothesis).
